# Xylem specific activation of 5’ upstream regulatory region of two *NAC* transcription factors (*MusaVND6* and *MusaVND7*) in banana is regulated by *SNBE*-like sites

**DOI:** 10.1371/journal.pone.0192852

**Published:** 2018-02-13

**Authors:** Sanjana Negi, Himanshu Tak, T. R. Ganapathi

**Affiliations:** 1 Plant Cell Culture Technology Section, Nuclear Agriculture and Biotechnology Division, Bhabha Atomic Research Centre, Trombay, Mumbai, India; 2 Homi Bhabha National Institute, AnushaktiNagar, Mumbai, India; National Taiwan University, TAIWAN

## Abstract

Deposition of secondary cell wall in the xylem elements is controlled by a subgroup of NAC (NAM, ATAF, CUC) family, known as vascular-related NAC transcription factors (VNDs). In the present study, we analyzed the 5’ upstream regulatory region of two banana NAC transcription factors (*MusaVND6* and *MusaVND7*) for tissue specific expression and presence of 19-bp *secondary-wall NAC binding element* (*SNBE*)-like motifs. Transgenic banana plants of *Musa* cultivar Rasthali harboring either *P*_*MusaVND7*_::*GUS* or *P*_*MusaVND6*_::*GUS* showed specific GUS (β-D-Glucuronidase) activity in cells of the xylem tissue. Approximately 1.2kb promoter region of either *MusaVND6* or *MusaVND7* showed presence of at least two *SNBE*-like motifs. This 1.2kb promoter region was retarded in a gel shift assay by three banana VND protein (VND1,VND2 and VND3). The banana VND1-VND3 could also retard the mobility of isolated *SNBE*-like motifs of *MusaVND6* or *MusaVND7* in a gel shift assay. Transcript levels of *MusaVND6* and *MusaVND7* were elevated in transgenic banana overexpressing either banana *VND1*, *VND2* or *VND3*. Present study suggested a probable regulation of banana *VND6* and *VND7* expression through direct interaction of banana VND1- VND3 with *SNBE*-like motifs. Our study also indicated two promoter elements for possible utilization in cell wall modifications in plants especially banana, which is being recently considered as a potential biofuel crop.

## Introduction

Evolution of secondary cell wall deposition was a prominent feature which not only provided the mechanical strength required for the vertical growth of plants but also helped in the long distance transport of water and minerals. This secondary cell wall is laid in cells of xylem tissue and is composed of cellulose, hemicelluloses, lignin and xylan. Water transport in plants from roots to other organs is through the channels of xylem tissue composed of tracheids and vessel elements. These tracheids develop an array of secondary wall depositions in the form of reticulate, pitted, helical and annular thickenings. Secondary cell wall deposition is regulated and highly coordinated for systematic deposition of multiple components by many genes among which a subgroup of *NAC* transcription factors, *VNDs* (*vascular related NAC transcription factors*) are most important. Secondary cell wall deposition in tracheids is accompanied with programmed cell death, hence regulation of the activity of secondary cell wall associated genes in specialized cells is of prime importance for proper functioning and homeostasis of plants.

Our earlier work identified seven *VND* (*VND1-VND7*) genes from banana genomic database and analyzed their expression pattern during the time course of *invitro* tracheary element differentiation from banana embryogenic cells in a brassinolide supplemented medium [1]. During tracheary element formation, transcript of banana *VND1-VND3* and *VND6-VND7* were detected as early as third day after induction with brassinolide [1]. Overexpression of banana *VND1-VND3* (*MusaVND1*, *MusaVND2* and *MusaVND3*) genes in transgenic banana indicated ectopic secondary wall deposition and tracheids development suggesting the important roles of these genes in tracheary element formation [2,3].

In *Arabidopsis*, roles of *vascular related NAC domain transcription factors* (*VND1-VND7*) in secondary wall deposition have been documented in detail [4–5].A pioneering work carried out by Kubo et al. (2005) showed that *ArabidopsisVND6* and *VND7* are master regulators of secondary wall deposition as their overexpression resulted in transdifferentiation of cells into tracheids [6]. However recent work on *VND1-VND5* from *Arabidopsis* has established that these genes also carry the potential to independently trigger the secondary wall deposition and tracheid differentiation [4]. Apart from *VND1-VND7*, other members of *NAC* group involved in secondary wall deposition have also been characterized.SECONDARY WALL ASSOCIATED NAC DOMAIN PROTEIN1 (SND1) and NAC SECONDARY WALL THICKENING PROMOTING FACTOR 1 (NST1)are independent master regulators of secondary wall deposition and functions redundantly in differentiation of xylem fibers in *Arabidopsis*[7–8]. Secondary wall thickening in anther endothecium isregulated by *NAC SECONDARY WALL THICKENING PROMOTING FACTOR 1 (NST1)*and *NST2*and T-DNA insertion lines for *NST1* and *NST2* are defective in anther dehiscence [9].

A common motif in the promoters of secondary wall associated genes for their regulation has been identified recently which has been named as *secondary-wall NAC binding element* (*SNBE*)- motif [10,11]. Further, some secondary wall associated transcription factors, *VND6*, *VND7* and *SND1* have been shown to regulate the expression of their downstream target genes by *SNBE*-like motif [10,11]. The sequence of *SNBE* motif has been worked out as an imperfect palindrome of 19bp with consensus sequence *(T/A)NN(C/T)(T/C/G)TNNNNNNNA(A/C)GN(A/C/T) (A/T)* and has been identified in the 5’upstream regulatory regions of multiple downstream target genes [10].*ArabidopsisSND1*, *VND6*, *VND7*, *NST1* and *NST2* have been shown to regulate the expression of downstream genes like *MYB46*, *MYB83*, *MYB103*, *SND3* and *KNAT7* through such *SNBE*-like motifs in their promoter region [10]. Furthermore, *ArabidopsisSND1*, *VND6* and *VND7* also regulate the expression of programmed cell death related gene like *XCP1*(tracheary elements related *cysteine proteases*) through direct binding to *SNBE* motif [10]. These findings suggest that *SNBE*-like motifs are common regulatory *cis*-element for direct regulation of secondary wall associated genes. Recently a potential regulation of *ArabidopsisVND7* expression was shown by binding of *ArabidopsisVNDs* to *VND7* promoter and its*SNBE* motif [12].

Lignocellulosic biomass which is primarily composed of secondary cell wall is an important source of renewable energy, paper and pulp production [13]. Hence, genetic factors with potential to modulate the biochemical properties of lignocellulosic biomass can be of great importance in the field of renewable energy and pulp production. However, most of the studies conducted on regulation of lignocellulosic biomass by secondary wall associated *NAC* transcription factors has resulted in unwanted side effect of growth reduction, possibly due to transdifferentiation of cells into tracheary elements [7,8]. Hence, a tighter control over the expression of *VNDs* and preferably limiting their overexpression in vascular tissue of transgenic plants will provide a suitable way for desired modification of plant lignocellulosic biomass. Hence, characterization of promoter region with specific activation in xylem vessel elementis indispensable. This study has characterized two such regulatory regions, *P*_*MusaVND7*_ and *P*_*MusaVND6*_and demonstrated their xylem specific activation.

In this study we report the characterization of 5’upstream regulatory region of two banana *VNDs* (*MusaVND6* and *MusaVND7*). The tissue specific expression of these was identified by cloning their promoter region upstream of *GUS* and analyzing the transgenic banana plants by GUS staining. We analyzed sequence of *MusaVND6* and *MusaVND7*promoter and identified *SNBE*-like sites which indicated their potential regulation by this important *cis*-element. Our study demonstrated that banana VND1, VND2 and VND3 could bind to 5’upstream regulatory region (approximately 1.2 kb) of banana *VND6* and *VND7* in a gel shift assay and also to *SNBE*-like motifs (present in *P*_*MusaVND7*_ and *P*_*MusaVND6*_). Moreover, elevated transcript level of *MusaVND6* and *MusaVND7* were detected in the corm tissue of banana overexpressing either banana *VND1*, *VND2* or *VND3*. Banana VND1-VND3 could trigger the expression of *GUS* from *P*_*MusaVND7*_ and *P*_*MusaVND6*_ indicating that these transcription factors could activate the expression of *MusaVND6* and *MusaVND7*. The present work indicated a potential regulation of banana *VND6* and *VND7* transcription factors through *SNBE*-like motif and also identified two xylem specific regulatory regions which can serve aspotential DNA elements for xylem specific cell wall modification. This work will also enhance our present knowledge about the regulation of secondary wall thickening in plants especially in monocots.

## Material and methods

### PCR amplification of 5’ up-stream region and cloning upstream of *GUS*

Genomic DNA of banana cultivar Rasthali was isolated from leaf tissue usingGenElute Plant Genomic-DNA Miniprep-Kit (Sigma,USA) following the instruction provided with the kit. DNA specific primers were designed and used for PCR amplification using the commercially available PCR master mix (Thermofisher scientific) and purified genomic DNA as template. PCR was carried out as follows: 98°C(10min), 35-cycles of [98°C(1min), 58°C(1min), 72°C(1.5min)] followed by final extension of 72°C(20min). *P*_*MusaVND7*_ was amplified with the forward primer (AACTGCAGCCTCGAAAGGAAAAGGTCAT *Pst*I underlined) and reverse primer (AACCATGGTGTGGATTCACCTGGAGAC *Nco*I underlined) and further PCR purified. Forward primer (AATCTAGAAAAGCATTCATTCCACGTACT *Xba*I underlined) and reverse primer AACCATGGTCATGCGAAGTGATTTAAAGTG *Nco*I underlined) were used for amplification of *P*_*MusaVND6*._ Binary vector *pCAMBIA 1301* was digested with *Pst*I or *Xb*aI and *Nco*I (to release the *CaMV35S* promoter) and then used for ligation with *Pst*I/*Nco*I digested *P*_*MusaVND7*_ and *Xba*I/*Nco*I digested *P*_*MusaVND6*_ to generate *pCAMBIA1301- P*_*MusaVND7*_:: *GUS* and *pCAMBIA1301- P*_*MusaVND6*_:: *GUS* respectively. The ligation reaction was confirmed by digestion and sequencing of recombinant binary vector. The sequences generated have been deposited in the NCBI database as MF429830 (for *P*_*MusaVND7*_) and MF429831 (for *P*_*MusaVND6*_).

### DNA gel-shift assay (EMSA)

The *SNBE*-like sites identified from the *P*_*MusaVND7*_ and *P*_*MusaVND6*_were synthesized as complementary oligonucleotides and annealed together at 37°C after heating at 90°C in a thermal cycler machine. The oligonucleotides containing *SNBE*-like site from *P*_*MusaVND7*_ are 5’-AATTCGATAGCCTT*T**GG**CGT**GCACTCA**AAG**A**AA*TTGGT-3’; and 5’-ACCAATTTCTTTGAGTGCACGCCAAAGGCTATCGAATT-3’(19bp *SNBE*-like site spanning from -186 to -167 is italicized and consensus resides are underlined). Complementary oligonucleotides with *SNBE*-like site from *P*_*MusaVND6*_ are 5’-AATTCGATAGCCTT*A**GT**TGT**AGGGCTC**AAG**A**TT*TTGGT-3’ and 5’-ACCAAAATCTTGAGCCCTACAACTAAGGCTATCGAATT-3’ (19bp *SNBE*-like site spanning from -220 to -201 is italicized and consensus resides are underlined).The annealed oligonucleotides or the PCR amplified promoter fragments was incubated with either banana VND1, VND2 or VND3 protein in a EMSA buffer (10mM Tris-buffer, pH8, 5mM MgCl_2_ and 10mM KCl) for 20 minutes. The reaction was later resolved on a 1.5% agarose-gel to visualize the DNA-protein interaction. Mutated *SNBE-like*(*mSNBE*) oligonucleotides were annealed as mentioned above and utilized in EMSA reaction as discussed before. The sequences of oligonucleotides for *mSNBE* were 5’-CCTTCGGAAAGCACTCATTTAGGTTGG -3’ and 5’- CCAACCTAAATGAGTGCTTTCCGAAGG -3’(for -186 to -167 position *SNBE-*like site of *P*_*MusaVND7*_; consensus residues of *SNBE* site were mutated and are underlined). Sequence of oligonucleotides used for mutated *SNBE –like* site of *P*_*MusaVND6*_ were 5’-CCTTCGTAAAAGGGCTCTTTAGGTTGG-3’and 5’- CCAACCTAAAGAGCCCTTTTACGAAGG-3’ (consensus residues of *SNBE* site were mutated and are underlined).A 250bp DNA fragment (-287 to -37 upstream of transcription start site) spanning the *SNBE-*like site (-186 to -167) was PCR amplified from *P*_*MusaVND7*_ using oligonucleotides 5’-GAGCATGTGGAAACCATAGC-3’ and 5’-AAGAACATCCCATACTTGGAA-3’ and was utilized in gel shift assay with the VND1-VND3 proteins. Similarly a 224bp DNA fragment (-388 to -164 upstream of transcription start site) encompassing the *SNBE-*like sites in *P*_*MusaVND6*_ was PCR amplified (using oligonucleotides 5’-CATCACCTTCTGTGGTATTCTTC -3’ and 5’- AGAGGAGCAACAAGTGATTGA -3’)for gel shift assay with VND1-VND3 proteins. EMSA reaction with these DNA was carried out as discussed above.

### Quantitative RT-PCR analysis

Transgenic banana cultivar overexpressing *MusaVND1*(KT356872),*MusaVND2* (KP335047) or *MusaVND3* (KP666170) have been described earlier by us[2,3]. Tissue of three independent plants was mixed in equal amount and then utilized for RNA isolation. Total RNA was isolated from the leaves and corm of the banana plant using Concert plant RNA reagent (Invitrogen, USA) as described in the manufacturer’s protocol. Total RNA was then cleaned using RNA binding column of RNeasy plant mini kit (Qiagen, Germany) and genomic DNA contamination was removed using on-column DNAase-digestion (Qiagen, Cat. No.79254) set as per the protocol supplied in the kit. Total RNA was analyzed on 1% agarose gel and then utilized for first strand cDNA synthesis using thermoscript AMV-RT (Invitrogen: Cat. No.12236-014).Sequence of the primers utilized are: FP 5’-AGGGATGGGTTGTGTGTAGG-3’ and RP 5’-TGCAGGTGGAGAAGATCTGG-3’ for *MusaVND6* (GSMUA_Achr8T11590_001); FP 5’-AGAGCAAAGCGAGTGGTACT-3’ and RP 5’-ACCCATCCTTCTTCCTGAGC-3’ for *MusaVND7* (GSMUA_Achr3T22360_001); FP 5’-CCGATTGTGCTGTCCTCATT-3’ and RP 5’-TTGGCACGAAAGGAATCTTCT-3’ for *MusaEF1α* (GSMUA_Achr6T02020_001). Transcript analysis was carried out on a rotor gene Q platform (Qiagen, Germany) using 2x SYBR Green Jump Start Taq Ready Mix (Sigma, USA) with 1:50 diluted cDNA. Reaction carried out under following conditions: 95°C(4min), 32-cycles of 95°C(15sec), 60°C(20sec), 72°C(20sec) ending with a melting curve analysis step. The C_t_-values were normalized with the expression of housekeeping gene *MusaEF1α* and fold change in expression of *MusaVND6* and *MusaVND7* was calculated by comparative Ct method (2^−ΔΔCt^) as described earlier [14].

### Transformation and generation of transgenic banana plants harboring either *P*_*MusaVND7*_:: *GUS* or *P*_*MusaVND6*_:: *GUS*

Binary vectors (*pCAMBIA1301- P*_*MusaVND7*_:: *GUS* and *pCAMBIA1301- P*_*MusaVND6*_:: *GUS*) were used for transformation of *Agrobacterium* strain *EHA105* [15] by electroporation. The recombinant colonies of *EHA105* were selected on LB-agar plate supplemented with kanamycin (50μg/ml) at 27°C in an incubator for 3 days. The recombinant *EHA105* was confirmed by colony PCR for presence of the binary vector. Overnight grown *Agrobacterium* was cultivated to OD of 0.6 and then induced by acetosyringone for 3 hours. Induced *Agrobacterium* was utilized for transformation of embryogenic cell suspension of banana cultivar Rasthali as described previously [16]. Banana embryogenic cells (0.5mlpacked cell volume for each co-cultivation tube) were co-cultivated with induced *Agrobacterium* for 30 minutes in presence of acetosyringone. Complete co-cultivation medium was aspirated under pressure upon glass fiber filtersusing a filter assembly. The glass fiber filters with banana embryogenic cells were then cultured in dark for three days on M2-medium supplemented with acetosyringone. Further, the cells were scrapped from the filters and the transformed cells were then selected for hygromycin resistanceafter culturing on banana embryo development medium (BEM) supplemented with cefotaxime (400mg/l) and hygromycin (5mg/l) as described earlier [16]. Developing embryos were cultured on fresh BEM with cefotaxime (400mg/l) and hygromycin (5mg/l) every 3 weeks until well developed embryos were visible (usually after 3 months of culture). Well developed embryos with potential to transform into shootswere converted into banana shootby culturing on embryo germination medium supplemented with 6-benzylaminopurine (0.5mg/l BAP) [16]. Putatively transformed banana shoots were then multiplied on banana multiplication medium (MS-medium with 2mg/l BAP and 30mg/l adenine sulphate). Rooting of the banana shoots were carried on MS-medium supplemented with NAA (1mg/l). Well rooted banana plants were hardened in sterile soil in a contained green house facility. Genomic DNA of transgenic banana lines was isolated from leaf tissue with the help of GenElute Plant Genomic-DNA Miniprep-Kit (Sigma,USA) following the instructions provided with the kit. The genomic DNA was utilized for PCR analysis of T-DNA integration in these banana plants. PCR was carried out for amplification of *promoter-GUS* fusion (~3.2kb) and promoter alone (~1.2kb for *P*_*MusaVND7*_and ~1.1 kb for *P*_*MusaVND6*_). For *promoter-GUS* fusion amplification, forward primer of either *P*_*MusaVND6*_and *P*_*MusaVND7*_and a reverse primer (CACGTGGTGGTGGTGGTGGTGGCTA) in *GUS* coding region was utilized while for amplification of either *P*_*MusaVND6*_and *P*_*MusaVND7*,_ primers utilized for their cloning were used. PCR running condition was 98°C(10min), 35-cycles of [98°C(1min), 58°C(1min), 72°C(3min)] followed by final extension of 72°C(20min).

### GUS staining of the histological sections and GUS activity analysis

The activation of *P*_*MusaVND7*_ and *P*_*MusaVND6*_in different organ and tissue was carried out by staining for GUS (β-D-Glucuronidase) activity. GUS staining buffer was prepared as follows: 1mM X-Gluc (5-Bromo-4-chloro-3-indolyl Glucuronide), 0.1% triton X-100, 0.5mM potassium ferricyanide and 0.5mM potassium ferrocyanide prepared in 100mM sodium phosphate buffer with pH 7.0. GUS staining was carried out at 37°C for maximum of 12 hours. Before recording the observations, chlorophyll of green tissue was removed by repeated incubation in 70% ethanol at 37°C till satisfactory resultwas obtained. Image of GUS stained sections of different organ was observed and recorded in Eclipse 80i, Nikon microscope.Estimation of GUS activity was carried out as picomoles 4-MU per min per μg soluble protein according to protocoldescribed earlier using 4-methylumbelliferyl-b-D-glucuronide (MUG) and soluble extract of plant [17].

### Transcriptional activation assay of *P*_*MusaVND7*_and *P*_*MusaVND6*_

The activation of *P*_*MusaVND7*_and *P*_*MusaVND6*_by VND1-VND3 was assayed in terms of GUS activity in banana embryogenic cells. Construct *pCAMBIA1301- P*_*MusaVND7*_:: *GUS* and *pCAMBIA1301- P*_*MusaVND6*_:: *GUS* generated above were utilized as reporter construct. *MusaVND1*, *MusaVND2* and *MusaVND3*were cloned in *pCAMBIA1302* in place of *GFP*utilizing the restriction sites *Nco*I and *Pml*I. Primers utilized are: FP 5’-AAATCATGAAATCGTGTGTTCCTCCT -3’ and RP 5’- AACACGTGTCATTGCTCAAATATACAGATGC-3’ for *MusaVND1*; FP 5’-AATCATGAAATCGTGTGTTCCTC -3’ and RP 5’-AACACGTGTCACTGCTCGAATATACAGATG -3’ for *MusaVND2*; FP 5’-AATCATGAATGCATTCCCTCATGT -3’ and RP 5’-AACACGTGTCATTTCCACAGTTCGACTT -3’ for *MusaVND3*(*Bsp*HI and *Pml*I sites underlined). *P*_*CaMV35S*_:*MusaVND1*, *P*_*CaMV35S*_:*MusaVND2* and*P*_*CaMV35S*_:*MusaVND3* served as effector constructs. *Agrobacterium* strain *EHA105* harboring different reporter and effector constructswere used for transient co-transformation of embryogenic cells of banana cultivar Rasthali as described earlier [3]. The cells aspirated onto glass fiber filters were analyzed for the GUS activity after fifth day of transformation. Banana cells transformed with either of the reporter construct served as control. For each replication 0.5ml packed cell volume was used for transformation and complete cells on one filter was utilized for GUS estimation. The experiment was repeated atleast three times.

## Results

### Isolation and sequence analysis of *P*_*MusaVND7*_and *P*_*MusaVND6*_

Expression of both *MusaVND6* and *MusaVND7*was elevated along with other genes during an *invitro* xylogenesis of banana embryogenic cell suspension in brassinolide supplemented medium indicating that they are important *NAC* transcription factors for tracheary element differentiation in banana [1]. 5-upstream region of the *MusaVND6* (GSMUA_Achr8T11590_001) and *MusaVND7* (GSMUA_Achr3T22360_001) were identified from the banana genome database (http://banana-genome-hub.southgreen.fr/) and primers were designed for PCR amplification of at least 1kb DNA region utilizing genomic DNA of banana cultivar Rasthali as template. These sequences were designated as *P*_*MusaVND7*_(1237 bp) and *P*_*MusaVND6*_(1143 bp) and were deposited in the NCBI database. Putative TATA box and transcription start site (+1 TSS) was determined by *insilico* analysis at TSSplant server (http://www.softberry.com/berry.phtml?topic=tssplant&group=programs&subgroup=promoter)Sequence analysis of either *P*_*MusaVND7*_ or *P*_*MusaVND6*_ revealed presence of at least two 19bp perfect *SNBE*-like sequences which were identified by the consensus sequence of *SNBE* site[*(T/A)NN(C/T)(T/C/G)TNNNNNNNA(A/C)GN(A/C/T) (A/T)*] reported earlier [10]. In *P*_*MusaVND7*_ first *SNBE*-like site (TGGCGTGCACTCAAAGAAA on plus strand; consensus residues are underlined) was positioned at -186 to -167 while the second *SNBE*-like site (TATCGTACGTAGTACGCTT on complementary strand; consensus residues are underlined) was positioned at -926 to -907 (S1 Fig). In case of *P*_*MusaVND6*_ first *SNBE*-like site was detected at -220 to -201 (AGTTGTAGGGCTCAAGATT on complementary strand; consensus residues are underlined) and second *SNBE-like* site was positioned at -372 to -353(TCCCTTTACACAGAAGAAT on complementary strand; consensus residues are underlined) relative to transcription start site (S2 Fig).

### Banana VND1-VND3 proteins bind to *P*_*MusaVND7*_ and *P*_*MusaVND6*_

**Fig 1:**
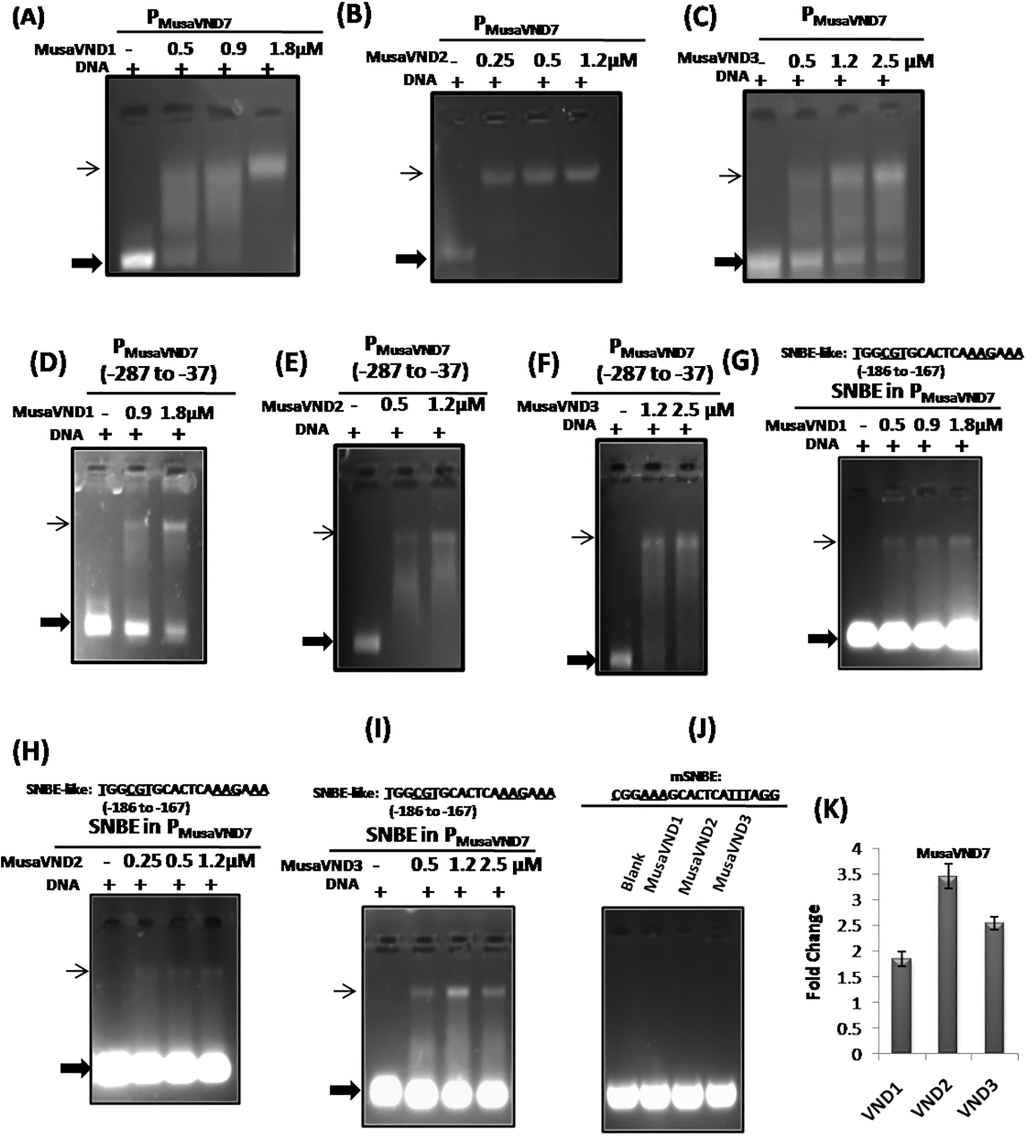
Regulation of *P*_*MusaVND7*_by banana VND1-VND3. (A-C) Retardation of *P*_*MusaVND7*_(approximately 1.2 kb region) by banana VND1- VND3 protein. The amount of protein utilized is indicated on the top of the figure. + sign indicates the presence of equal amount of DNA in each reaction. (D-F) Retardation of a 250 bp *P*_*MusaVND7*_ fragment (-287 to -37 relative to transcription start site) containing the *SNBE*–like site by banana VND1- VND3 protein. The amount of protein utilized is indicated on the top of the figure. + sign indicatesthe presence of equal amount of DNA in each reaction.(G-I) *SNBE*–like site in the *P*_*MusaVND7*_ (-186 to -167) was used for gel shift analysis with banana VND1- VND3 protein. The conserved residues in the *SNBE* site (19bp) are underlined. The *SNBE*-like site was retarded in the gel by VND1- VND3 protein. Thin arrow indicates the retarded DNA as protein-DNA complex while thick arrow indicates the unbound DNA. (J) Banana VND1-VND3 failed to bind the mutated *SNBE-like* site (*mSNBE*). The mutated residues in *SNBE –like* site are underlined. (K) Transcript level of *MusaVND7* in the corm tissue of the transgenic banana overexpressing either banana *VND1*, *VND2* or *VND3*. Fold change in control was kept at one and the data was normalized by the expression of banana *EF1α*.

**Fig 2:**
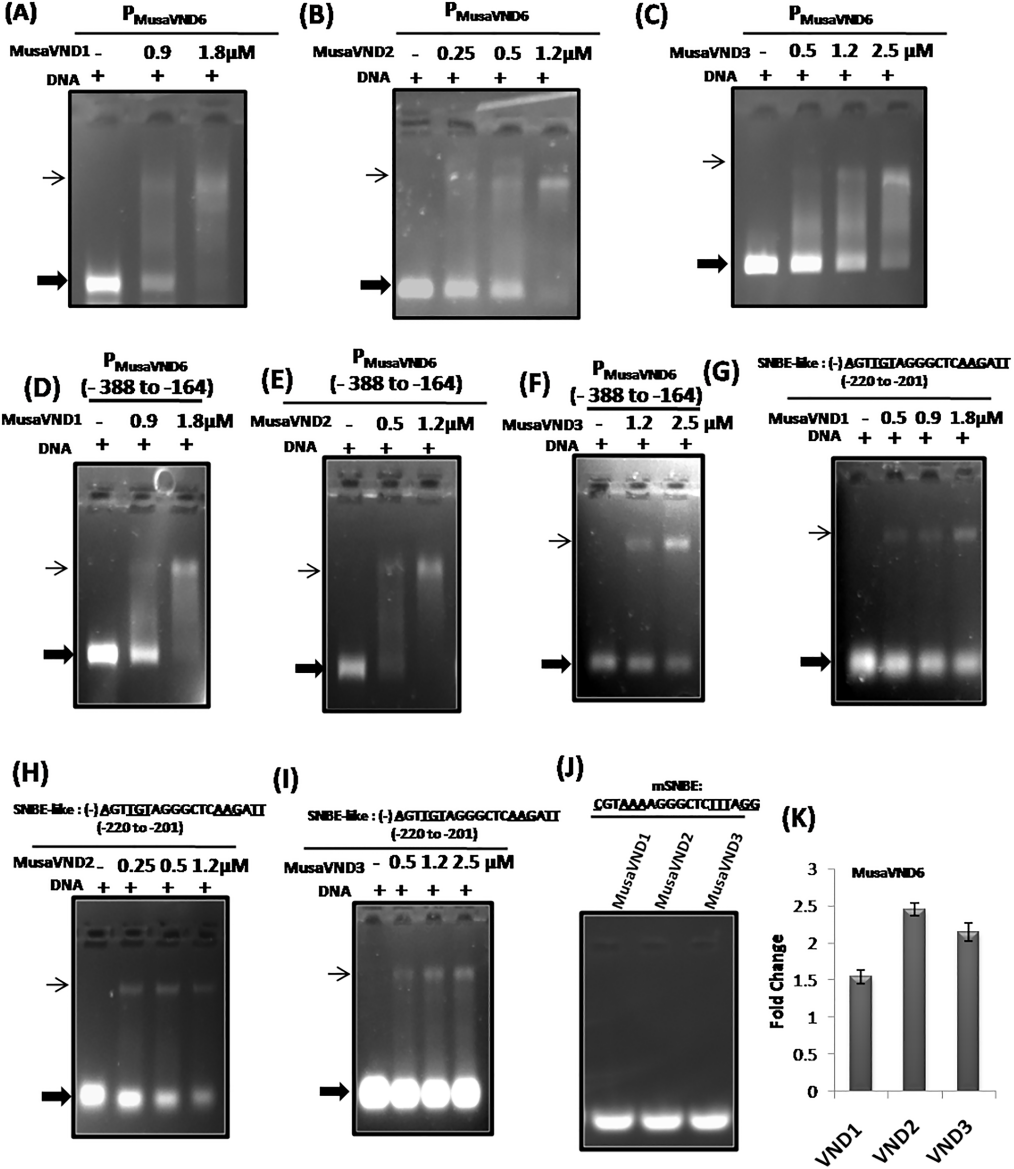
EMSA analysis of *P*_*MusaVND6*_ with banana VND1-VND3. (A-C) The *P*_*MusaVND6*_(~1.2 kb) was amplified from genomic DNA of banana cultivar Rasthali and incubated with the indicated amount of the protein (either banana VND1, VND2 or VND3). The amount of the retarded DNA (thin arrow) increased with the increasing amount of the protein. Presence of equal amount of DNA in each reaction is indicated by +signs. (D-F) Retardation of a 224 bp *P*_*MusaVND6*_ fragment (-388 to -164 relative to transcription start site) containing the *SNBE*–like sites by banana VND1- VND3 protein. The amount of protein utilized is indicated on the top of the figure. + sign indicates the presence of equal amount of DNA in each reaction. (G-I) *SNBE*–like site (-220 to -201) detected in the *P*_*MusaVND6*_was retarded in a gel shift assay by VND1- VND3 protein. The minus sign indicates the presence of *SNBE* site on the complementary strand. Thin arrow indicates the retarded DNA as protein-DNA complex while thick arrow indicates the unbound DNA. (J) Banana VND1-VND3 failed to bind the mutated *SNBE-like* site (*mSNBE*). The mutated residues in *SNBE –like* site are underlined. (K) Quantitative RT-PCR analysis of *MusaVND6* in the corm tissue of transgenic banana overexpressing either of banana *VND1*, *VND2* or *VND3*. Fold change in control was kept at one and the data was normalized by the expression of banana *EF1α*.

Vascular related NAC domain containing proteins(VNDs) are reported to regulate the expression of genes by binding to the *SNBE*-motifs in the promoter region[10]. Moreover, *Arabidopsis* VND1-VND7 proteinsare shown to bind *SNBE* site in isolation or as a part of *ArabidopsisVND7* promoter for its potential regulation [12]. We also analyzed whether banana VND proteins have similar potential to recognize the *SNBE* site of *P*_*MusaVND7*_ and *P*_*MusaVND6*_in a gel shift assay. For this a 1.2 kb 5’upstream region of banana *VND7* was incubated with different concentration of banana VND1-VND3 proteins in appropriate reaction condition and the interaction was analyzed after resolving on a gel. Mobility of *P*_*MusaVND7*_ in gel shift assay was retarded by all the three banana VNDs (VND1, VND2 and VND3) proteinsuggesting that *P*_*MusaVND7*_ is a potential target gene for regulation by banana VND1- VND3 (Fig 1A, 1B and 1C). Similarlyfor analyzing whether, banana VND1- VND3 could recognize and bind the 5’upstream region of banana *VND6*, a 1.1kb region of *P*_*MusaVND6*_was incubated with different concentration of either banana VND1, VND2 or VND3 protein in EMSA buffer and the interaction was visualized after resolving the reaction on a gel. Our results indicated that banana VND1- VND3 could retard the mobility of a 1.1kb region of *P*_*MusaVND6*_indicating that *P*_*MusaVND6*_is also a potential gene regulated by banana VND1- VND3 (Fig 2A, 2B and 2C). We also checked whether banana VND1-VND3 could retard a small fragment of *P*_*MusaVND7*_ and *P*_*MusaVND6*_with*SNBE-like* site.Results indicated that VND1-VND3 were able to retard a 250bp *P*_*MusaVND7*_ fragment (-287 to -37 relative to transcription start site) containing the *SNBE*–like site present at -186 to -167 (Fig 1D, 1E and 1F). Similarly the mobilityof a 224bp *P*_*MusaVND6*_ fragment (-388 to -164 relative to transcription start site) encompassing the *SNBE*–like sites was retarded by banana VND1- VND3 protein in gel shift assay (Fig 2D, 2E and 2F). These results suggested that *P*_*MusaVND7*_ and *P*_*MusaVND6*_ are potential target genes for regulation by VND1-VND3.

### Banana VND1-VND3 proteins binds to *SNBE*-like sequences of *P*_*MusaVND7*_ and *P*_*MusaVND6*_

As *SNBE*-like sitewas detected in both *P*_*MusaVND7*_ and *P*_*MusaVND6*_and mobility of *P*_*MusaVND7*_ and *P*_*MusaVND6*_was retarded by VND1- VND3, we analyzed whether VND1- VND3 could bind to these *SNBE*-like sites. A 38bp double stranded DNA containing *SNBE*-like site of *P*_*MusaVND7*_ (TGGCGTGCACTCAAAGAAA positioned at -186 to -167) was incubated with either banana VND1- VND3 protein and the interaction was analyzed on a gel. All the three VND1- VND3 strongly retard the mobility of *SNBE*-like site of *P*_*MusaVND7*_ indicating that banana VND1- VND3 recognize the *SNBE*-site in *P*_*MusaVND7*_(Fig 1G, 1H and 1I). Similarly the *SNBE*-like site in *P*_*MusaVND6*_was also bound by banana VND1- VND3 in a gel shift assay indicating that banana VND1- VND3 bind*SNBE*-like site in *P*_*MusaVND6*_(Fig 2G, 2H and 2I). However, banana VND1-VND3 failed to bind a mutated version of the *SNBE-*likesite (*mSNBE* where the consensus residues of *SNBE* site were mutated) in *P*_*MusaVND7*_ and *P*_*MusaVND6*_ indicating that VND1-VND3 specifically interact with *P*_*MusaVND7*_ and *P*_*MusaVND6*_ and the interaction is mediated by *SNBE-like* sites (Figs 1J and 2J).

### Overexpression of banana *VND1-VND3* induce *MusaVND6* and *MusaVND7*

As the banana VND1- VND3 could bind the ~1.2kb of *P*_*MusaVND7*_ and *P*_*MusaVND6*_ as well as *SNBE*-like site within them, suggesting expression of these genes may be altered after overexpression of either *VND1*, *VND2* or *VND3*. We analyzed the expression of *MusaVND6* and *MusaVND7* in leaves and corm tissue of transgenic plants overexpressing either banana *VND1-VND3* by quantitative RT-PCR [2,3]. Results indicated that expression of both *MusaVND7* and *MusaVND6* was elevated in corm tissue of transgenic banana plants overexpressing either banana *VND1*, *VND2* or *VND3* (Figs 1K and 2K). These results suggested that banana *VND1-VND3* regulate the expression of banana *VND6-VND7* through *SNBE*-like motifs.

### Banana VND1-VND3 activate expression from *P*_*MusaVND7*_ and *P*_*MusaVND6*_

**Fig 3:**
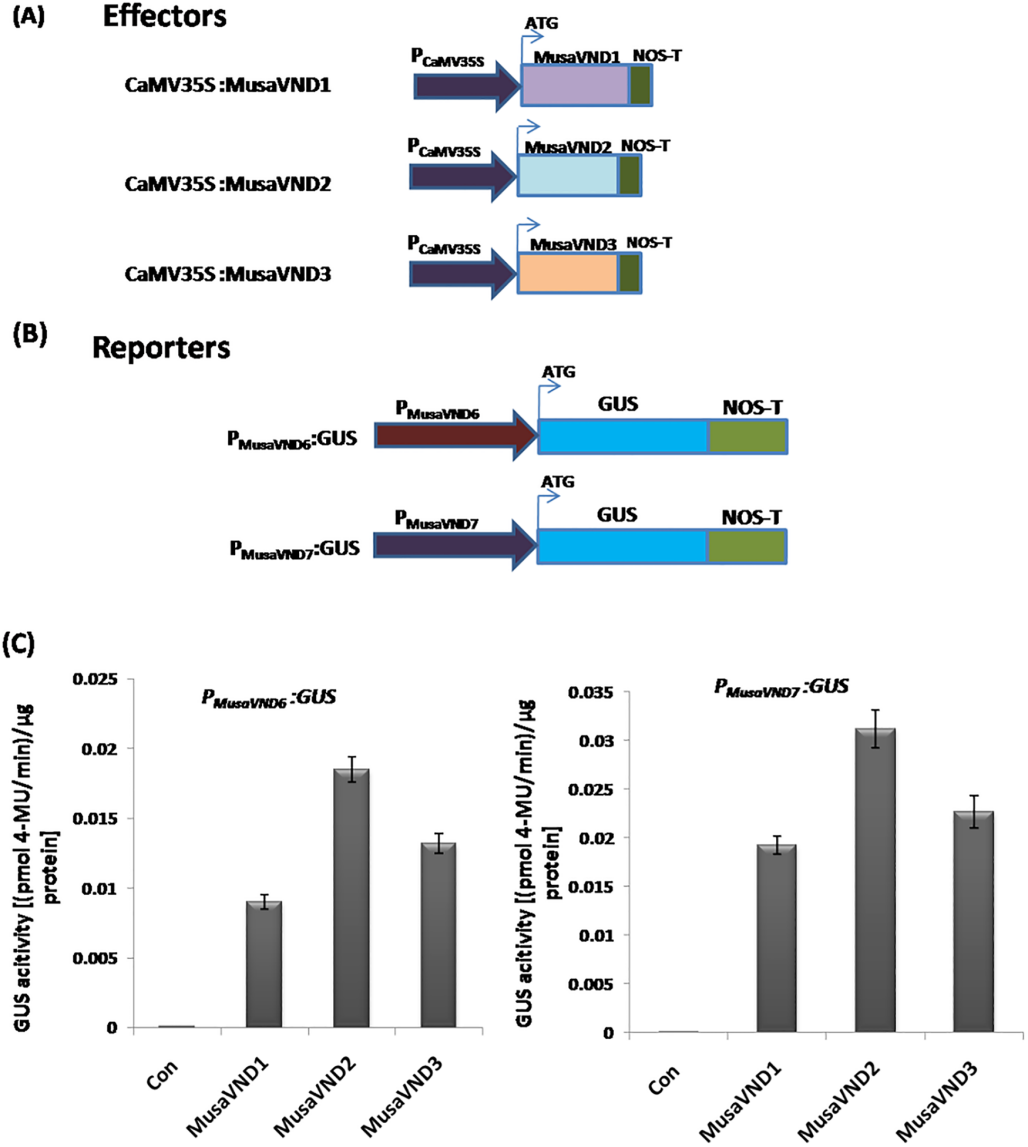
Transcriptional activation assay. VND1-VND3 induce the *VND6* and *VND7* promoter. (A) Diagrammatic representation of the effector constructs used for the assay. The *VND1*-*VND3* coding sequence were driven by the *CaMV35S* promoter. (B) Different reporter constructs employed for the assay. *P*_*MusaVND7*_ and *P*_*MusaVND6*_ fused upstream of *GUS* in *pCAMBIA1301* served as reporter constructs. (C) GUS activity obtained after fifth day of transient activation assay. Data was represented as mean±SD of three replication and represented as (pmol 4-MU/min)/μg protein.

To test whether the elevated transcript levels of *MusaVND6* and *MusaVND7* in transgenic banana overexpressing VND1-VND3 is due to transcriptional activation of *P*_*MusaVND7*_ and *P*_*MusaVND6*_ by VND1-VND3, we performed a transient activation assay using *GUS* as reporter gene. Effector constructs contain *CaMV35S* promoter driving the expression of VND1-VND3 (Fig 3A). Reporter constructs harbor *P*_*MusaVND7*_ and *P*_*MusaVND6*_ fused upstream of *GUS* coding sequence (Fig 3B).Different combination of effector and reporter constructs were utilized for analyzing the transcriptional activation of *P*_*MusaVND7*_ and *P*_*MusaVND6*_ by VND1-VND3 and the GUS activity was estimated after fifth day of the transient co-transformation. Data obtained indicated that transformation of banana embryogenic cell suspension with reporter and effector constructs (containing VND1-VND3) remarkably elevated the GUS activity (Fig 3C). Cells transformed with reporter alone showed negligible GUS activity as *P*_*MusaVND7*_ and *P*_*MusaVND6*_are inactive in embryogenic cells of banana.

### Generationand molecular analysis of banana plants harboring either *P*_*MusaVND7*_::*GUS*, *P*_*MusaVND6*_::*GUS*

Vascular related *NAC* domain genes are regulator of secondary wall deposition and xylem element development and *VND1-VND3* regulate the expression of banana *VND6*-*VND7*, we analyzed the tissue specific activation of *P*_*MusaVND7*_ and *P*_*MusaVND6*_. Both *P*_*MusaVND7*_ and *P*_*MusaVND6*_ were cloned upstream of *GUS* in *pCAMBIA1301* and transgenic banana plants harboring either *P*_*MusaVND7*_::*GUS*, *P*_*MusaVND6*_::*GUS* were generated. *Agrobacterium* harboring binary vector*pCAMBIA1301- P*_*MusaVND7*_:: *GUS* and *pCAMBIA1301- P*_*MusaVND6*_:: *GUS*was used for transformation of banana embryogenic cells of cultivar Rasthali and transformed cells were selected in culture medium supplemented with 5mg/l hygromycin (S3A and S4A Figs). Growth of transformed cells on embryo development medium resulted in emergence of white and opaque somatic embryos (S3B and S4B Figs). Developing somatic embryos showed different stageof development like globular to torpedo shaped stage and further resulted in emergence of secondary embryos indicating appropriate development pathway toward generation of banana plants (S3C and S4C Figs). Transgenic banana plants were cultured on banana multiplication medium for clonal propagation (S3D and S4D Figs) and individual shoots were separated and rooted on rooting medium (S3E and S4E Figs). Appropriately rooted banana plants harboring either *P*_*MusaVND7*_::*GUS*, *P*_*MusaVND6*_::*GUS*was hardened in a contained green house for further analysis (S3F and S4F Figs).The T-DNA insertion in these transgenic banana plants was analyzed by PCR amplification of either *P*_*MusaVND7*_*-GUS* fusion or *P*_*MusaVND6*_*-GUS* fusion and *P*_*MusaVND7*_ or *P*_*MusaVND6*_from the leaf genomic DNA. PCR analysis showed positive amplification of *promoter-GUS* fusion product and promoter alone indicating successful integration of *P*_*MusaVND7*_::*GUS*and*P*_*MusaVND6*_::*GUS* in genome of these banana plants (S5A and S5B Fig).These plants were then analyzed for tissue specific activity of GUS controlled by activation of *P*_*MusaVND7*_ or *P*_*MusaVND6*_.

### Xylem specific activation of 5’-upstream regulatory region of *MusaVND*7

**Fig 4:**
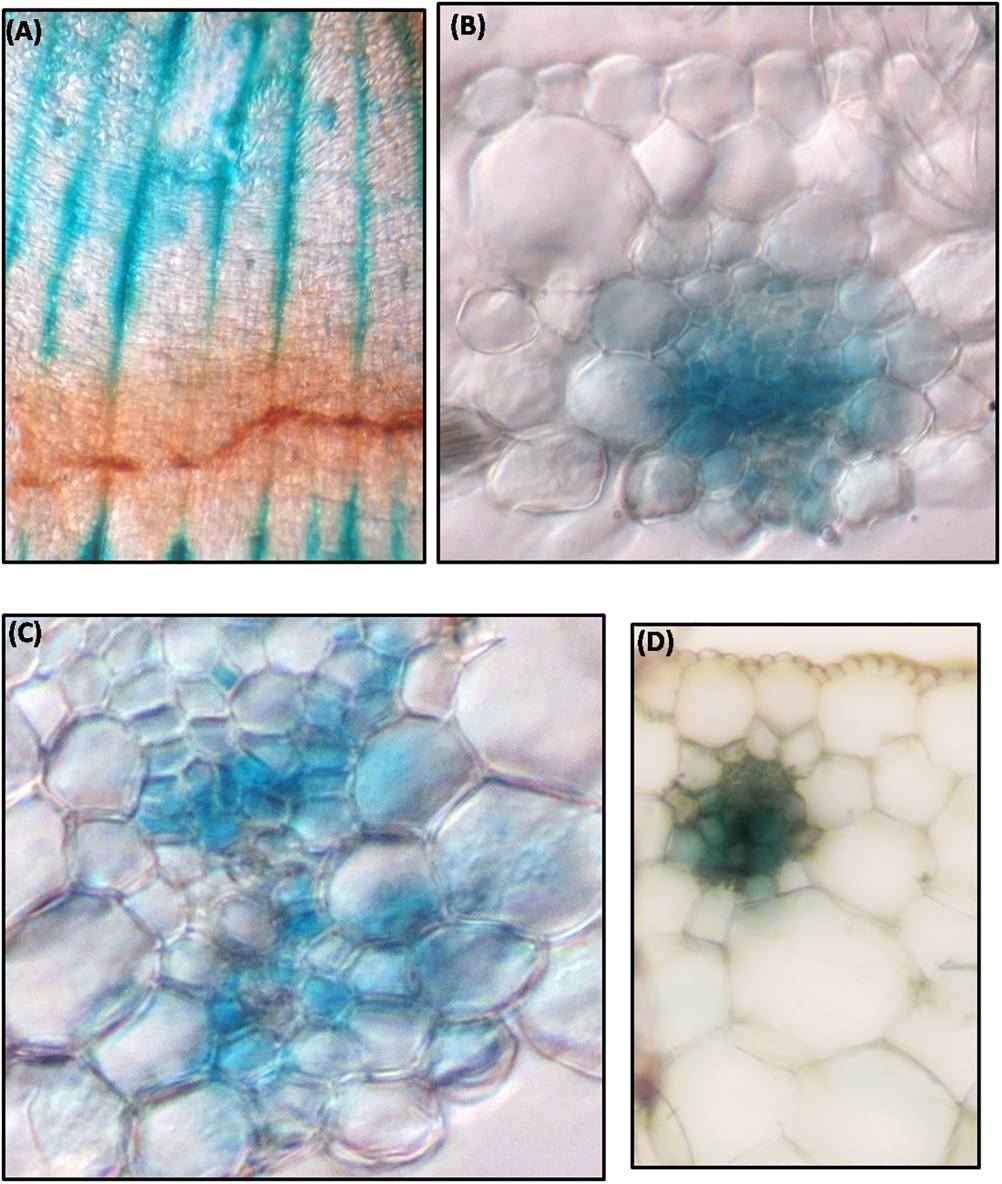
Expression analysis of *GUS* under the control of *P*_*MusaVND7*_ in leaves and petiole. (A) GUS staining observed in banana leaf due to activity of *P*_*MusaVND7*_. (B) Transverse section of GUS stained leaf showing prominent and specific GUS staining in cells of the vascular bundle. Note the absence of staining in other cells. (C) Close-up of one of the leaf vascular bundle showing GUS staining in vessels and tracheids of the xylem. (D) Transverse section of the petiole indicating the vascular specific activity of the *MusaVND7* promoter. Note the GUS activity in the central vessel cells and surrounding tracheary elements.

**Fig 5:**
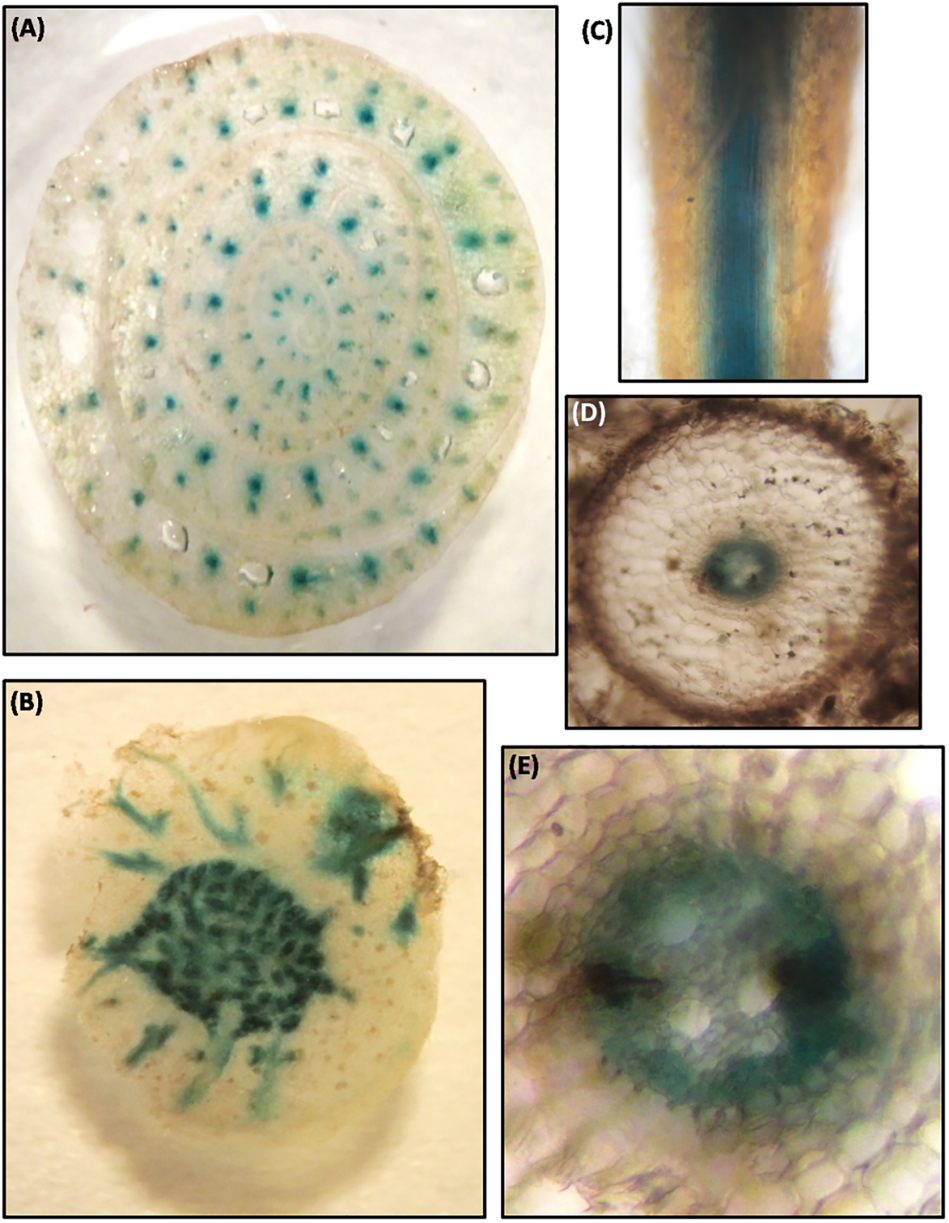
Activity of *MusaVND7* promoter in pseudostem, corm and roots of banana. (A) Cross section of the pseudostem demonstrating the vascular bundle specific activation of the *P*_*MusaVND7*_. Note the absence of GUS staining from tissues other than vascular bundles of the pseudostem. (B) Activity of *MusaVND7* promoter in corm of the banana. *P*_*MusaVND7*_got activated only in vascular tissues. Note the specific and intense GUS activity in vascular strands of the central cylinder. Also note the GUS activity in the vascular strands traversing from central vascular cylinder to the outwards growing roots. (C) GUS staining observed in the central vascular cylinder in the roots of banana harboring *P*_*MusaVND7*_:: *GUS*. (D) Cross section of the GUS stained root. (E) Close-up view of the central vascular cylinder showing activation of *P*_*MusaVND7*_in protoxylem as well as metaxylem vessels and surrounding tracheids.

Tissue specific activation of *P*_*MusaVND7*_ was ascertained by analyzing the expression of *GUS*in banana plants harboring the *P*_*MusaVND7*_::*GUS*. At least three independent transgenic lines were analyzed and all of them showed similar GUS expression pattern and representative resultwas shown. GUS staining of leaf tissue showed *GUS* expression in longitudinal veins as well as in commissural veins (Fig 4A). Analysis of transverse section of the leaf suggested prominent *GUS* expression in vascular bundles while other tissues do not show any GUS staining indicating very specific activation of *P*_*MusaVND7*_ in xylem tissue (Fig 4B). Close-up of one of the leaf vascular bundle showed evidently strong GUS expression in xylem vessels and surrounding tracheary elements (Fig 4C). Similarly transverse section of the petiole was analyzed which also indicated vascular specific activation of *P*_*MusaVND7*_ as *GUS* expression was precisely observed in vessels and tracheids (Fig 4D). In pseudostem, *GUS* expression was observed in all the vascular bundle of different leaf sheath and moreover, the *GUS* expression appeared to increase from inner to outer sheath indicating that*P*_*MusaVND7*_ activation increased with age suggesting important role of *MusaVND7* in vascular tissue development (Fig 5A). In corm, specific and intense GUS activity was observed in vascular strands of the central stele region as well as in the vascular strands traversing from central vascular cylinder to the outwards growing roots (Fig 5B). GUS staining of the whole roots displayed *GUS* expression in the central cylinder (Fig 5C and 5D) and in cross section, the *GUS* expression appeared to predominate in the protoxylem and metaxylem vessels as well as nearby tracheids (Fig 5E). These results indicated that *MusaVND7* showed xylem specific expression and is a probable important regulator of xylem development.

### 5’-upstream sequence of *MusaVND6* is active in vascular tissues

**Fig 6:**
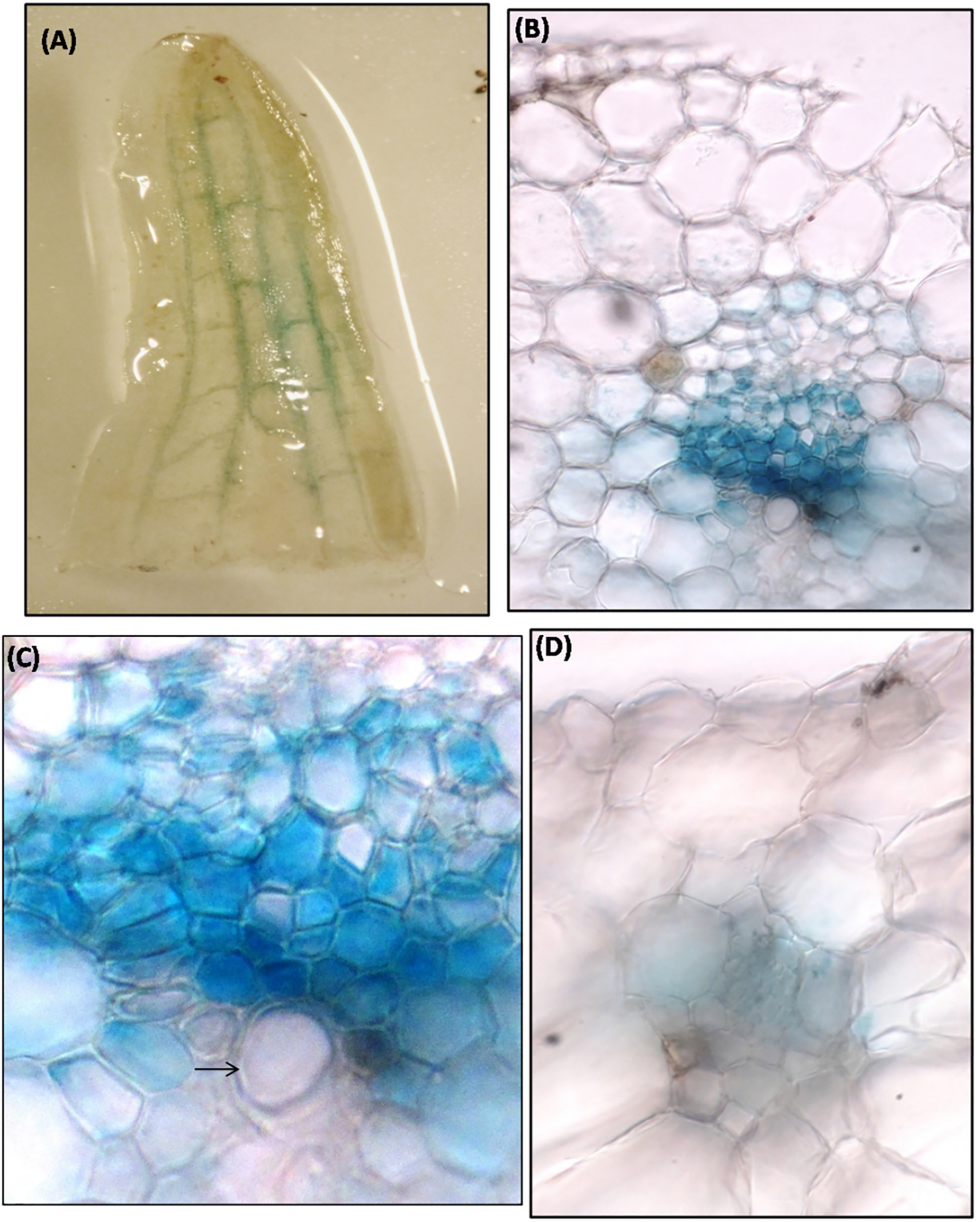
Analysis of activation of *P*_*MusaVND6*_in leaf and petiole of transgenic banana harboring *P*_*MusaVND6*_:: *GUS*. (A) GUS staining observed in veins of banana leaf due to activation of *P*_*MusaVND6*_. (B) Transverse section of the leaf showing specific activation of the *P*_*MusaVND6*_ in cells of the vascular tissue. Note the absence of the staining from the other tissue of the leaf. (C) Close-up of the leaf vascular bundle showing the GUS activity in the tracheids surrounding the vessel element (indicate by black arrow). (D) GUS staining in the petiole of banana. Note the GUS staining in vascular bundle.

**Fig 7:**
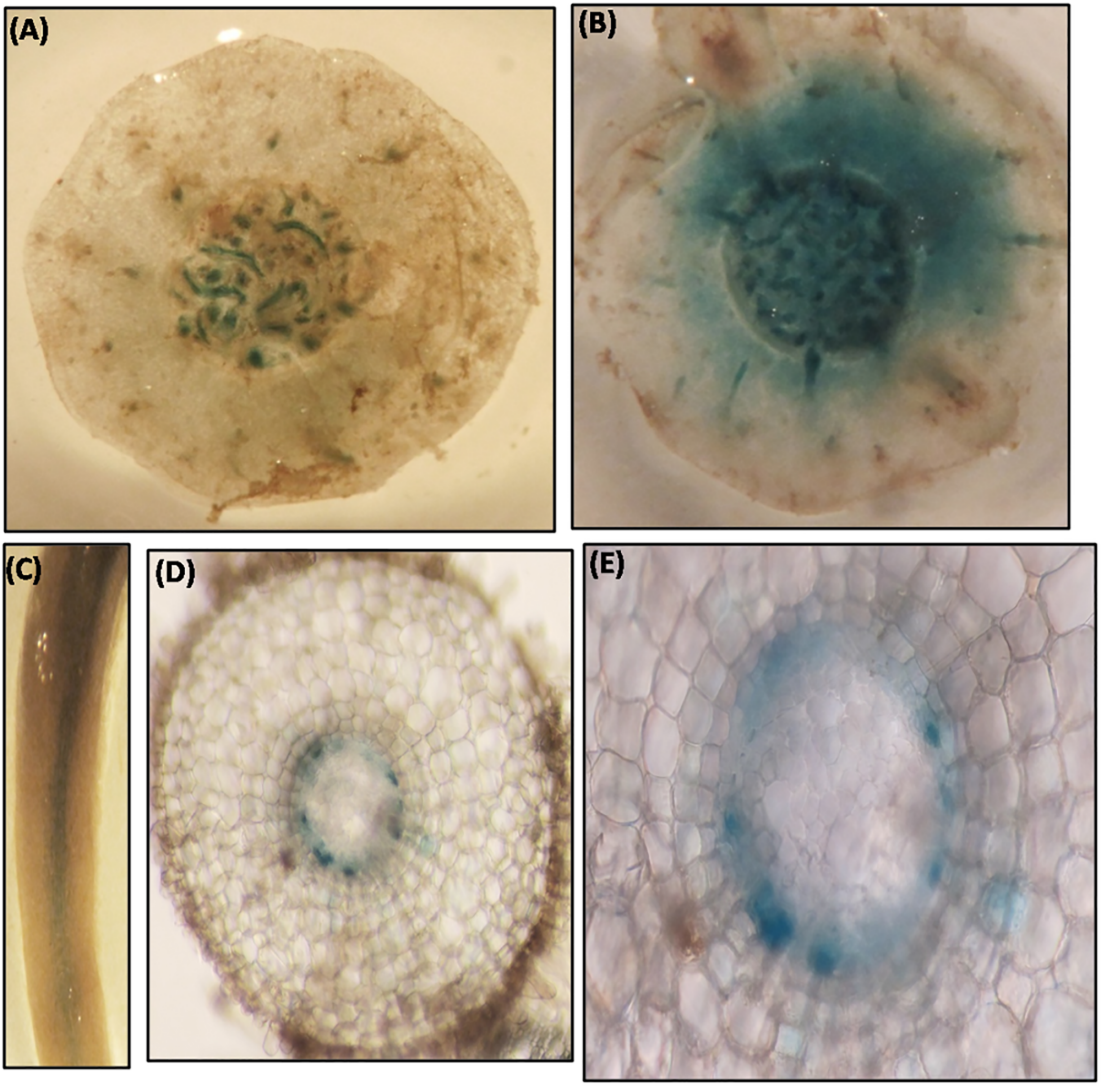
GUS activity in the pseudostem, corm, and roots of the banana harboring *P*_*MusaVND6*_:: *GUS*. (A) Transverse section of the pseudostem displaying the GUS activity in the vascular strands. (B) GUS staining observed in the corm of the banana. (C) Root of transgenic banana harboring *P*_*MusaVND6*_:: *GUS* showing GUS activity in the central vascular region. (D) Transverse section of the root showing specific GUS staining in the central vascular region. (E) Close-up of central vascular cylinder. Note the GUS staining in the peripheral protoxylem elements while it is absent from the central metaxylem region.

Transgenic banana harboring *P*_*MusaVND6*_::*GUS* were generated to analyze the tissue specific expression pattern of *MusaVND6*. Three independent transgenic lines showed similar *GUS* expression pattern and representative images were recorded. *GUS* expression in leaves was specifically observed in longitudinal veins (Fig 6A) which was confirmed by GUS staining of transverse section which showed explicit and strong *GUS* expression in vascular bundles with no *GUS* expression in epidermal or cortical cells (Fig 6B). In leaves vascular bundle, the *GUS* expression was prominent in tracheary elements surrounding the xylem vessel (Fig 6C). GUS staining of the petiole showed activation of *P*_*MusaVND6*_ in cells of vascular bundle while it was undetectable in other tissue typeindicating important role of *MusaVND6* in xylem development (Fig 6D). In pseudostem, activation of *MusaVND6* promoter was observed in central cylinder, and scattered vascular bundles of leaf sheaths (Fig 7A). In corm the activation of *P*_*MusaVND6*_ was observed in vascular bundles of central stele however, GUS staining was also observed in cells of the cortical region suggesting a probable differential activation of *MusaVND6* in different organs (Fig 7B). In roots the *GUS* expression was observed in central vascular region (Fig 7C and 7D) however, the *GUS* expression was prominent in peripheral protoxylem elements while it is absent from the central metaxylem region (Fig 7E). These results suggested that *MusaVND6* have important role in xylem development.

### GUS activity under the control of *P*_*MusaVND6*_and*P*_*MusaVND7*_

**Fig 8:**
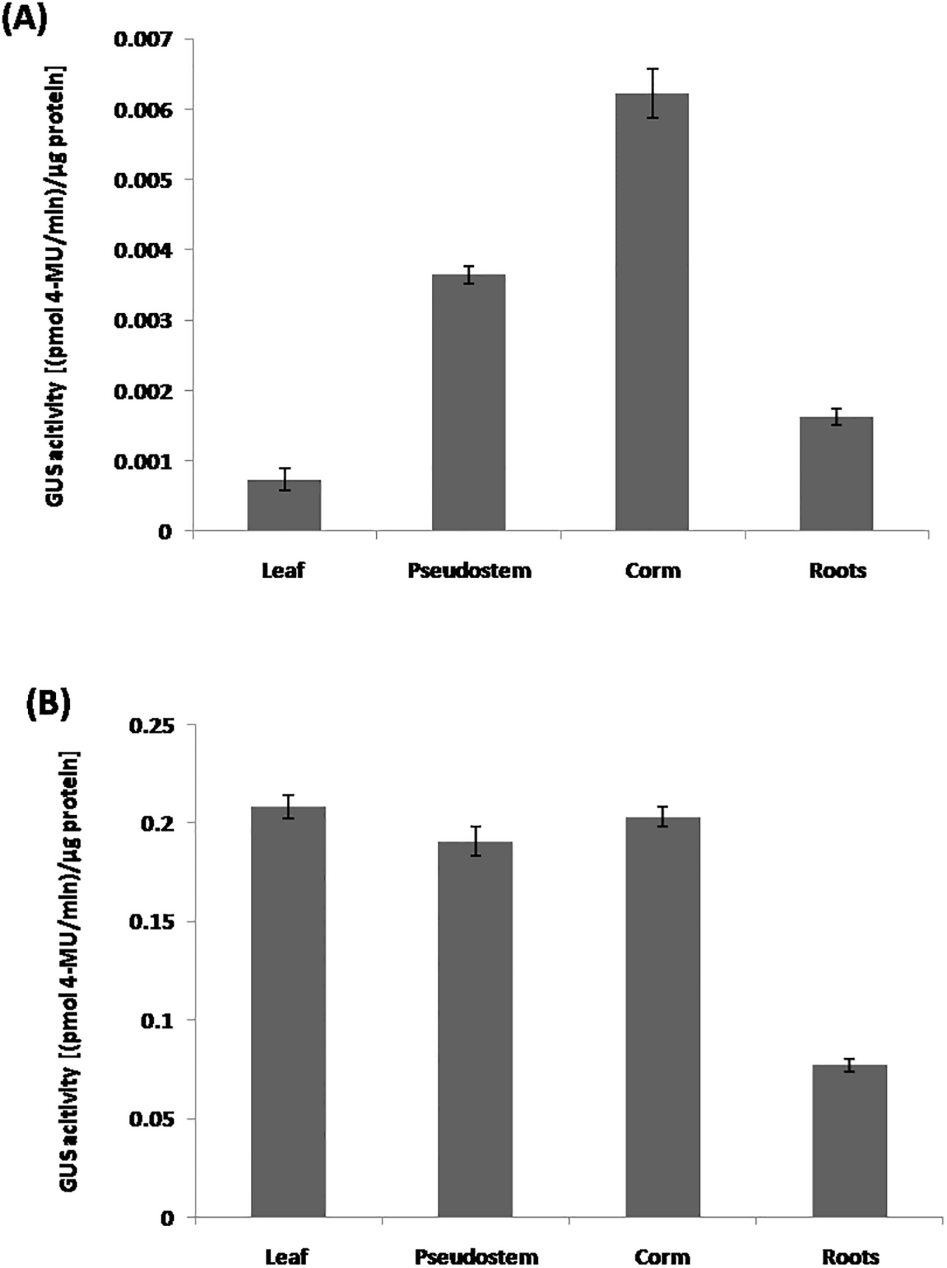
GUS activity in banana due to *P*_*MusaVND6*_:: *GUS* or *P*_*MusaVND7*_:: *GUS*. (A) GUS estimation in different organ of transgenic banana transformed with *P*_*MusaVND6*_:: *GUS*. (B) GUS activity estimated by fluorometric analysis in different organ of banana harboring *P*_*MusaVND7*_:: *GUS*. Data was represented as mean±SD of three replication and represented as (pmol 4-MU/min)/μg protein.

We estimated the GUS activity in various organs of banana harboring either *P*_*MusaVND6*_:: *GUS* or *P*_*MusaVND7*_:: *GUS* to quantitate the tissue specific activation of *P*_*MusaVND6*_and *P*_*MusaVND7*_. *P*_*MusaVND6*_and *P*_*MusaVND7*_ could drive the *GUS* expression in all the organs of the banana albeit at different level suggesting qualitative difference in the activity of these promoter in banana organs. *P*_*MusaVND6*_drives highest *GUS* expression in banana corm and pseudostem followed by roots and leaves (Fig 8A). While the extent of GUS activation by *P*_*MusaVND7*_ was almost similar in leaves, pseudostem and corm of transgenic banana and lower activityof GUS was observed in root tissue compared to other organs(Fig 8B).

## Discussion

Modifications of secondary cell wall including reduction of lignin especially in plants important for biofuel and pulp production holds great importance. The efficient conversion of lignocellulosic biomass (chiefly made up of secondary cell wall) into ethanol through fermentation process is deterred by high lignin content [18]. Hence reduction of lignin and increased deposition of hydrolysable cellulose through elevated deposition of secondary cell wall can be achieved by*NAC* transcription factors and other potential genetic factors. Moreover, many important crop plants like banana are prone to lodging during fruiting stage (due to heavy bunch weight), which can be improved through increased mechanical strength of pseudostem by elevatingsecondary cell wall deposition [19,20]. Information on regulation of secondary wall thickening especially in context of *VNDs* in plants like *Arabidopsis*, poplar and *Brachypodium* among otherhave been generated suggesting that these transcription factors regulate a number of downstream genes for coordinated regulation of secondary cell wall deposition [21–24]. In *Arabidopsis* seven members have been included in *VND* subgroup of *NAC* family and *VND6* and *VND7*are suggested to be master regulators for secondary wall synthesis [5,6]. Attempts to overexpress these genes for secondary cell wall modification have often resulted in unwanted effect of biomass reduction probably through transdifferentiation of essential tissue into tracheids as these genes were overexpressed under constitutive promoter [5,7]. Hence, identification and characterization of potential xylem specific regulatory DNA element for regulated expression of *VNDs* is indispensable [18]. In this study we have characterized two 5’upstream regulatory region (*P*_*MusaVND7*_ and *P*_*MusaVND6*_) with specific activation in xylem tissue element which can be utilized in future for biomass engineering. Some examples of utilization of xylem specific promoter element have been reported in the past. In *Arabidopsis*, the expression of *C4H* (*cinnamate 4-hydroxylase*), an important gene for lignin synthesis was restricted under the activation of *VND6* promoter (a vessel specific promoter) which reduced the lignin content (without plant growth reduction), thus increasing the hydrolysis of cellulose for sugar release [18]. Another study has reduced lignin content through expression of bacterial *3-dehydroshikimate dehydratase* (*QsuB*) under the *P*_*C4H*_and emphasized the need of fiber cell specific promoter for higher stringency in lignin reduction [25]. Moreover, studies on genetic factors regulating secondary wall deposition in banana are important due to the fact that banana has recently being researched as a potential second generation biofuel crop due to high amount of lignocellulosic biomass being left out after the fruit harvest [26–28]. Quantitative RT-PCR analysis to determine the transcript levels of *VND6* and *VND7* in native banana indicated differential expression of *MusaVND6* and *MusaVND7* in different organs. Transcript levels of both *VND6* and *VND7*was high in corm and pseudostem compared to other organs (S6 Fig). Though the transcript level of *VND7*was higher than *VND6* in different organ, but it does not correlate with the relative GUS activity observed in transgenic banana harboring*P*_*MusaVND7*_::*GUS* and *P*_*MusaVND6*_::*GUS*. Lower GUS activity in transgenic banana harboring *P*_*MusaVND6*_::*GUS* than transgenic banana harboring *P*_*MusaVND7*_::*GUS* might be due to reasons such as integration of T-DNA in less active chromatin regions or requirement of additional upstream DNA element for achievement of full potential inexpression of *VND6*. In the present study we have successfully analyzed the xylem specific activity of *P*_*MusaVND6*_and *P*_*MusaVND7*._

Expression pattern of the seven *VND*genes during tracheary element differentiation was different suggesting that these genes may function in different aspects of tracheid development [1,6]. Multiple secondary wall associated transcription factors like *VNDs* and *SND1* regulate downstream genes by binding to a 19bp imperfect palindrome known as *SNBE*-like motif and its consensus sequence was worked out to be *(T/A)NN(C/T) (T/C/G)TNNNNNNNA(A/C)GN(A/C/T) (A/T*) suggesting a common mode of regulation of a set of genes involved in tracheids differentiation [10]. *ArabidopsisSND1* and *VND7* have been shown to regulate the expression of multiple class of genes like *MYB* transcription factors (*MYB46*, *MYB83*, *MYB58*/*72*, *MYB48*/*59*, *MYB52* and *MYB85*), cell wall associated genes like *IRX* genes (*irregular xylem*) and programmed cell death related genes like *XCP1/2* (*xylemcysteine peptidase*) through *SNBE*-like motifs [29–31]. Cross regulation of *VND* genes is relatively unknown, however, a recent work on analysis of *SNBE*-like motif in promoter of *ArabidopsisVND7* and its regulation by VND1-VND7 proteins suggested potential regulation of *VND7*through*SNBE*-like cis-elements besides other known and unknown mechanisms [12]. Some studies on regulation of *VND7* have been carried out suggesting multiple level of regulation of its expression.*VND-INTERACTING2* (*VNI2*), is a transcriptional repressor which binds with VND proteins including VND7 protein and repress the expression of the secondary wall associated genes directly controlled by *VND7* [32]. Amount of *Arabidopsis* VND7 in cells is regulated by proteasome mediated degradation as accumulation of VND7 protein was observed upon treatment of cells with MG-132,which is a potent proteasome inhibiting chemical [33]. Two *ASYMMETRIC LEAVES2* (*AS2)/LATERALORGANBOUNDARIESDOMAIN* (*LBD*) gene family members *ASL19*/*LBD30* and *ASL20*/*LBD18*were reported to regulate the expression of *VND6* and *VND7* via a positive feedback loop as the expression of *ASL19*/*LBD30* and *ASL20*/*LBD18* is dependent on VND6 and VND7 and plants expressing *P*_*ASL20*_:*ASL20-SRDX* showed reduced expression of *VND6* and *VND7* [34]. Recently other genetic factors like *GATA5*, *GATA12*, *ANAC075 LBD15/ASL11*are shown to induce the *VND7* promoter suggesting additional regulations over *VND7* expression [12].

Our work showed that banana *VND6* and *VND7* expression is positively regulated by banana VND1- VND3 protein through direct binding with*SNBE*-like motif in the *P*_*MusaVND7*_ and *P*_*MusaVND6*_.Our report is in line with study on *ArabidopsisVND7* wherein its promoter was identified and induced by VND1-VND7 proteins [12] suggesting cross regulation of *VND* genes among themselves. In our previous work, we had successfully purified banana VND1-VND3 proteins and demonstrated their regulation over multiple downstream target genes including *MYB*transcription factors and *IRX* genes by binding to *SNBE*-like sites in their regulatory region [35]. Our work also identified presence of *SNBE*-like sites in the regulatory regions of banana *VND6* and *VND7* prompting us to analyze whether these genes can also be regulated by other banana VNDs. Here we show that banana VND1- VND3 could also bind to *P*_*MusaVND7*_ and *P*_*MusaVND6*_ as well as *SNBE*-like motif in them using a agarose based gel shift assay [36]. The binding of the VND1-VND3 get abolished by mutating the *SNBE* consensus residues indicating that the interaction of these three transcription factors with the *P*_*MusaVND7*_ and *P*_*MusaVND6*_ is specific and is governed by the presence of *SNBE*-like motifs. Elevated transcript levels of *VND6* or *VND7*was detected in the corm but not in leaves tissue of transgenic banana overexpressing either *VND1*, *VND2* or *VND3*. Moreover results obtained from transcriptional activation assay indicated that banana VND1-VND3 could activate transcription from the *P*_*MusaVND6*_ and *P*_*MusaVND7*_ confirming that these transcription factors not only bind but also could activate the expression of both *MusaVND6* and *MusaVND7*.

Present work showed the specific activation of *P*_*MusaVND6*_ and *P*_*MusaVND7*_ in sclerenchymatous cells (xylem tissue) suggesting that these promoters are inactive in non-sclerenchymatous cells. Overexpression of *VND1*-*VND3* in banana transdifferentiate many non sclerenchymatous cells into sclerenchymatous type and our previous work has indicated that compared to other organs, corm tissue showed remarkably higher transdifferentiation [2,3]. Moreover, banana corm contain abundant xylem tissue compared to other organs suggesting that elevation of *VND6* and *VND7* in *VND1*-*VND3* overexpression plants probably occurred specifically in native sclerenchymatous as well as transdifferentiated sclerenchymatous cells of transgenic corm. Besides, there is a possibility of tissue specific regulation of *VND6* and *VND7* by VND1-VND3 as suggested by differential up regulation in transgenic banana overexpressing *VND1*-*VND3*. Previous work on regulation of *ArabidopsisVND7* by VND1-VND7 through *SNBE* motifs has suggested possible presence of some unknown inhibitory factors regulating the expression of *VND7* and possibly its transcript stability in non-sclerenchymatous cells of plants overexpressing *VND1*-*VND7* and thus restricting its expression [12]. Further studies on identification of these possible inhibitors and the probable differential regulation of *VND6*-*VND7* transcript are warranted.Besides studies on regulation of transcript levels of *VND6* and *VND7* due to expression of *VNDs* in non-xylem tissue need to be carried out in detail. Nonetheless this study identified two potential xylem specific promoter regionswhich will be useful for crop improvement and presented an interesting mechanism for regulation of *VND6* and *VND7*expression in banana.

## Supporting information

S1 Fig Sequence analysis of *P*_*MusaVND7*_. Sequence of 5’ upstream regulatory region of *MusaVND7*. The *P*_*MusaVND7*_was amplified from genomic DNA of *Musa* cultivar Rasthali and analyzed for the presence of *SNBE*-like sites. *SNBE*–like sites in the *P*_*MusaVND7*_are boxed in green. Putative TATA box is shown in red and the transcription start site (+1 TSS) is underlined.
(PDF)

S2 Fig Sequence analysis of *P*_*MusaVND6*_. Sequence of 5’ upstream regulatory region of *MusaVND6* from *Musa* cultivar Rasthali. TATA box is boxed in red and transcription start site (+1 TSS) is indicated by an underline. *SNBE*–like sites detected in the sequence are boxed in green.
(PDF)

S3 Fig Generation of transgenic banana harboring *P*_*MusaVND7*_:: *GUS*. (A) Embryogenic cells of banana cultivar Rasthali transformed with *pCAMBIA1301- P*_*MusaVND7*_:: *GUS* and growing on banana embryo development medium supplemented with hygromycin (5mg/l). (B) Emergence of putatively transformed embryos on hygromycin supplemented medium after 2 months of growth. (C) Close-up of embryos showing growth in various stages of development as well as emergence of secondary embryos. (D) Multiplication of transgenic shoots on banana shoot multiplication medium. (E) Rooting of transgenic banana plants on banana rooting medium containing NAA(1mg/l). (F) Rooted banana plants were hardened in a green house for GUS analysis.
(PDF)

S4 Fig Generation of transgenic banana harboring *P*_*MusaVND6*_:: *GUS*. (A) Embryogenic cells of banana cultivar Rasthali growing on banana embryo development medium supplemented with hygromycin (5mg/l) after transformation with *pCAMBIA1301- P*_*MusaVND6*_:: *GUS*. (B) White and opaque embryos developed from continuous culturing of the transformed embryogenic cells on embryo development medium. (C) Developing embryos appeared globular to torpedo shaped in close-up. (D) Transformed banana shoots were multiplied on shoot multiplication medium to generate multiple lines. (E) Rooting of transgenic banana plants on banana rooting medium containing NAA(1mg/l). (F) Rooted banana plants were hardened in a green house for GUS analysis.
(PDF)

S5 Fig PCR analysis of integration of T-DNA in genomic DNA of transgenic banana plants. (A) PCR analysis of banana plants harboring *P*_*MusaVND7*_::*GUS*. (B) PCR analysis of banana plants harboring *P*_*MusaVND6*_::*GUS*. Two different primer pairs were utilized in PCR analysis. 1: FP in either *P*_*MusaVND7*_ or *P*_*MusaVND6*_ and RP in GUS; 2–4:FP and RP in either *P*_*MusaVND7*_ or *P*_*MusaVND6*_ 2–4: analysis of three independent transgenic banana lines. M: 1 KB DNA ladder. 1kb and 3 kb band size are indicated. (FP: forward primer; RP: reverse primer).
(PDF)

S6 Fig Transcript abundance analysis of *MusaVND6* and *MusaVND7*. Transcript level of *MusaVND6* and *MusaVND7* in tissue of different organs of wild type banana was analyzed by quantitative RT-PCR. Expression of *VND6* and *VND7* in different tissue types is shown after normalization of the data by the expression of banana *EF1α*. Data was represented as mean±SD of three replications.
(PDF)
