# Supplementary material for: Xylem specific activation of 5’ upstream regulatory region of two NAC transcription factors (MusaVND6 and MusaVND7) in banana is regulated by SNBE-like sites
Source: PLoS One. 2018 Feb 13;13(2):e0192852. doi: 10.1371/journal.pone.0192852 (PMC5811034; doi:10.1371/journal.pone.0192852)
Supplement: S1 Fig — Sequence of 5’ upstream regulatory region of MusaVND7. The PMusaVND7was amplified from genomic DNA of Musa cultivar Rasthali and analyzed for the presence of SNBE-like sites. SNBE–like sites in the PMusaVND7are boxed in green. Putative TATA box is shown in red and the transcription start site (+1 TSS) is underlined. (PDF) [file pone.0192852.s001.pdf]

```
-1068 CCTCGAAAGG AAAAGGTCAT GATATTTTAT GTATATATGC ATACATCATA
-1018 CATATATATA ATCAACGCAT GGTCCTATCT TGGTGCATTA ATTATGTTCC
 -968 AAGTCATGGA ACTTCTGTTA TCCCTTCCCC TCCTGACTTA AATAGCATGC
 -918 ATCATGCGAA AAGCCAAGTG TTCTTTGTTT GTGCAAGCTT CAATTAATGA
 -868 TCCTTGTCCT GAAGCTAATC TTGGCCTGAA ACTGGACTGT GGACACGAAT
 -818 GATTGAGTGT ACGATGCAAG GCACATTCCC AGTGCACATA AAGCAACAAG
 -768 TGCCATAGGG TGGTCCTTCC TTTGGCTGCC TCCACATCTT GACTTCCAGA
 -718 CCAAGTAGCC TTGCAGCATA AAATAGCGGC GCAAAGGGGG GCTGTTGGTG
 -668 TGCATGAGAG AGGTGGCGGT GGCTTAAGAT TTCTTTCTTG CTGTTGCCCC
 -618 CACCGCCCAA ATCGCCCTCA GGCCACCCTC TCCTTCTCTC CTTTCCCGTT
 -568 CCTGTTTGAT CACTGCACGC GCACTCGATG GCTGCTAACC CCCCGTCTCT
 -518 TCCTTTCTTT ACATTCCTCC TTTTTATTCT TTGTCCTTCC ATCGCCTTCT
 -468 GAACTTTGTA TATAAGCCTG GACAGCCTCT AAACTAGCAT TTTCTGTCTC
 -418 TCACTTTCTC TCTTTCTCCT TCTGCACGCG AAGAAGCCTG GCTGCCGGTG
 -368 AGTTCCTCAA CATCTCAGAG TAAATTACGC TGTTCCGCAG AGCTTACTGT
 -318 GGTCGTACCG TCTTTCTCCT CCTCCACTGA GAGCATGTGG AAACCATAGC
 -268 TATTGTAGGT ATATGCTTCT CTCTTGCAGC CTTGGGAAAT CGAGGTGTGG
 -218 TGGTCACTGA AACTGAAAGC AGCCACATCA TTGGCGTGCA CTCAAAGAAA
 -168 CATAGGCATG ATTGAAATTT ATAGTCAATT TTTCCATCTG CTTTCTATGT
 -118 TATACTCACT AAGATGATCA GATCTAGAAC TTCGAAATAT ATTCCATACG
  -68 CTTGTTGAAT TCCAAGTATG GGATGTTCTT AATTATATAA AAGATTTAGA
  -18 ATGGGATATA ATGTAAATAG CATTACCTTC TTGCACTGAT GGTGTACCAG
                      +1 TSS
   33 GTCATTTTCC AATCAATTCC TCCTATTTGC TTTCTAGATT TTGGGGATCT
   83 CTTTGATGAG GGTCTAAATC TTGTAGGGCC CGCTCATCAC CTCTTCATCC
  133 ATCTTAACTT GGTGCGAGGT CTCCAGGTGA ATCCACA
```

Fig S1
