# Supplementary material for: Xylem specific activation of 5’ upstream regulatory region of two NAC transcription factors (MusaVND6 and MusaVND7) in banana is regulated by SNBE-like sites
Source: PLoS One. 2018 Feb 13;13(2):e0192852. doi: 10.1371/journal.pone.0192852 (PMC5811034; doi:10.1371/journal.pone.0192852)
Supplement: S2 Fig — Sequence of 5’ upstream regulatory region of MusaVND6 from Musa cultivar Rasthali. TATA box is boxed in red and transcription start site (+1 TSS) is indicated by an underline. SNBE–like sites detected in the sequence are boxed in green. (PDF) [file pone.0192852.s002.pdf]

```
-844 AAGCATTCAT TCCACGTACT TGGAACCTTT TCGCCAACCA GTCTCAGCAC
-794 TCTTCGGTAA CCAGTAAGAG CTATTCCTTC TCCCACTCTC ATCTCCCTCC
-744 CTTCCTCTCT CCACACACAC ACACACACAC ATAATTATGT TTCTGAGCTT
-694 GCATTTTCTG GTCACAGATC GAAGGTGATA CGATTTGTAT GATTTCATGA
-644 TATATCGGTA CATAATTGCG TCTTTATTGC ATGAAGAGCT TCGATCATCC
-594 CAGTCTTTTA AATGTTGATG TAAATCTTTA TGTTTGTTTG ATGCTTTCTA
-544 AAGCTTTTCT AGCTGATCAA GCCAATATTG TCAGAATACA TCTTTTCCTT
-494 CGCGGCCATC AACGATGAGA GATTACCTCA AGAACATGCC ATGAATTCAC
-444 TCTCTACATA TAATTTTTGT AACCTTTTGT GATTAAAATA TCGTTGTGTC
-394 TATCTTCATC ACCTTCTGTG GTATTCTTCT GTGTAAAGGG ACATTGTTGC
-344 TTTGTTCATG GGACGATCAC ATTAGTTCTC AGATAATTAA TATGCATTTA
-294 CGCACTTCGG TAGTCTGCAT ATTACAGCAT GTATAATTGC CTGGACTACC
-244 ATGTTCTTCT AATGACAAGG CAAGAATCTT GAGCCCTACA ACTAGGTTTT
-194 GAACATGAAT CAATCACTTG TTGCTCCTCT AATAGCAACA TTTATCCATT
-144 TCGCATGCTC TAATCCACAA ACAACATTTA TCCATTTTGT TTTGTTTATA
 -94 CCCATCTTAC AAAGCTGTGT ATGTTTATTT ACCTGTAGAC TACTATGTGA
 -44 AGTATCTCGG AATATAAAGC TCTTGCATCA TTTACCCATC TTTGAGCATT
                                                  +1 TSS
   7 TCTTGGAATG AGTCATTGAT TGTTCATTTA TTTGAGTCAG AGACAATGGT
  57 AGGTTCCTAA GAAGCTATGA ATCTTTTCCA AATAGCTCTT CTAATCTGAA
 107 CATTTGATGT CACCTCTCAC ACTCTTTACC TCCTCCTATG CATGGAGTCC
 157 TATTCAAATT CGATCTGGCA TTCCAATCTC AATACATGCT TCTGTTTGCT
 207 GGATCACTAC TCAAACACAA GCTCAGCAAA TGTCTAATTC TCCTGGAAGT
 257 CACATCTGCA GAACTTAACC ACACTTTAAA TCACTTCGCA TGA
```

Fig S2
