# Supplementary figures and images for: Xylem specific activation of 5’ upstream regulatory region of two NAC transcription factors (MusaVND6 and MusaVND7) in banana is regulated by SNBE-like sites
Source: PLoS One. 2018 Feb 13;13(2):e0192852. doi: 10.1371/journal.pone.0192852 (PMC5811034; doi:10.1371/journal.pone.0192852)

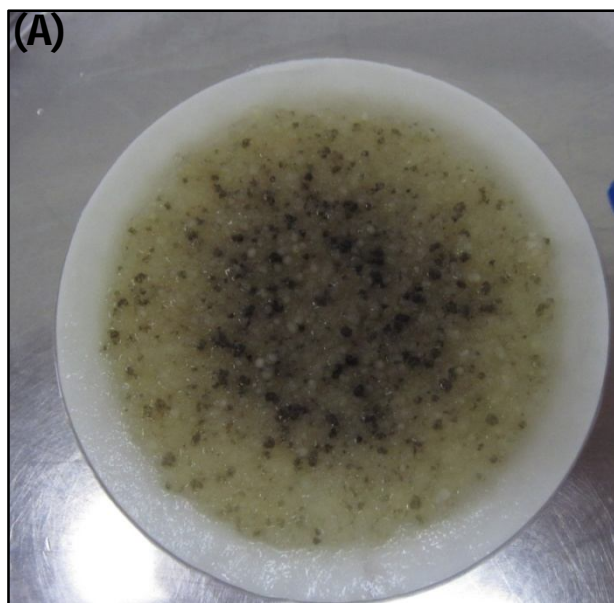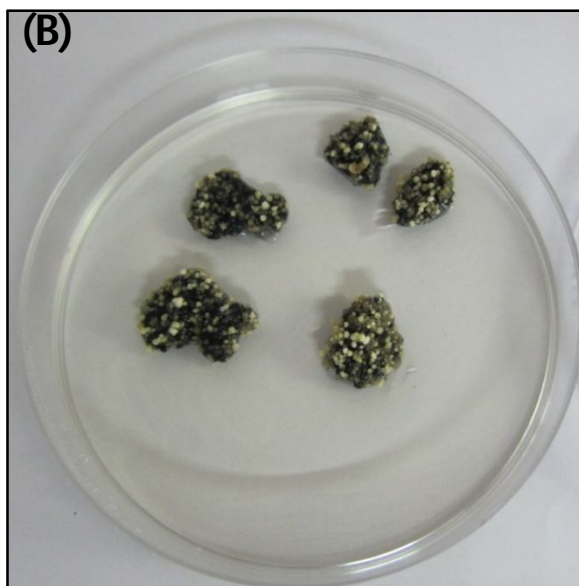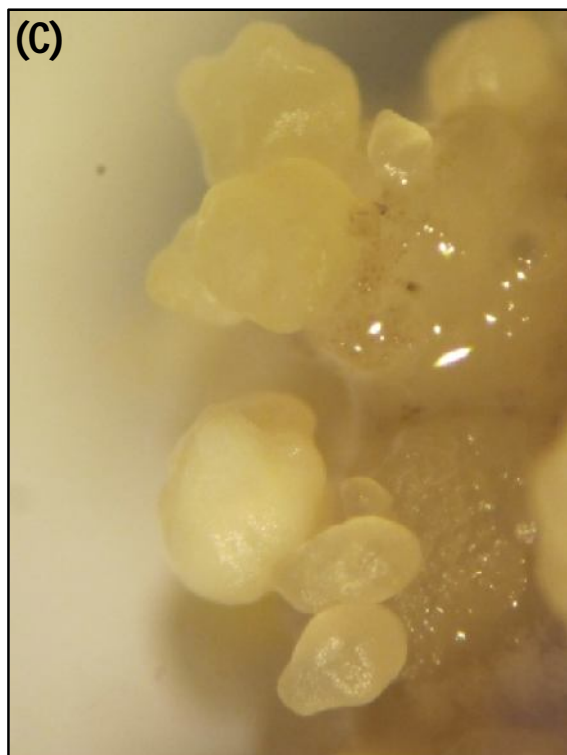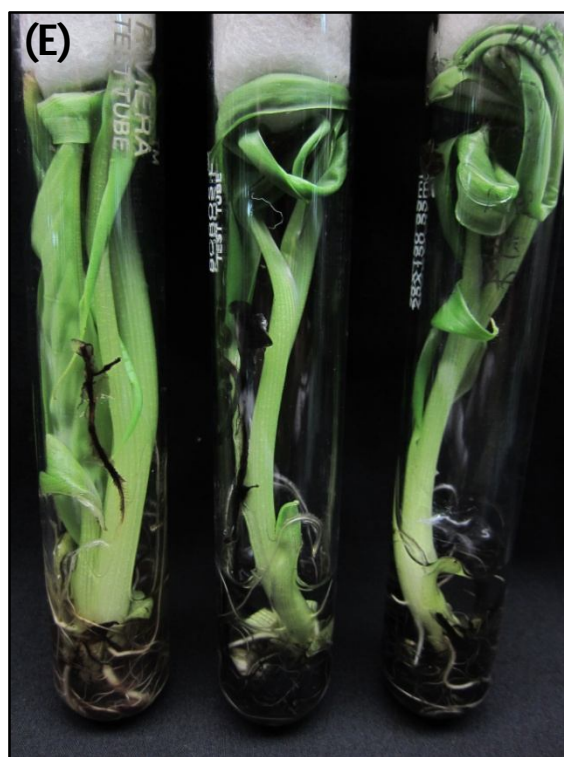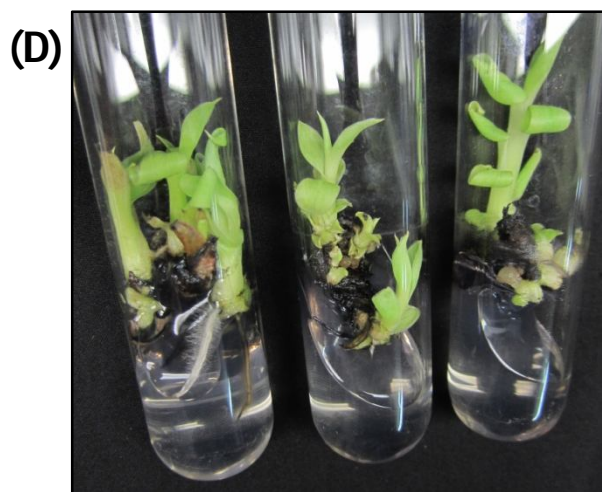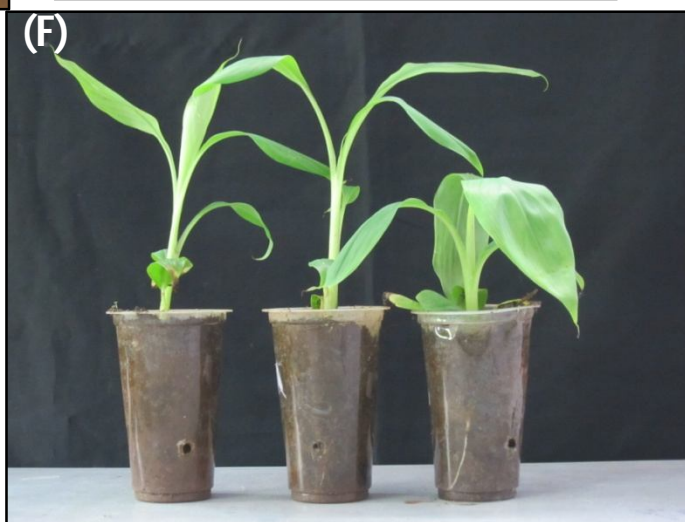

Fig S3

Supplement: S3 Fig — (A) Embryogenic cells of banana cultivar Rasthali transformed with pCAMBIA1301- PMusaVND7:: GUS and growing on banana embryo development medium supplemented with hygromycin (5mg/l). (B) Emergence of putatively transformed embryos on hygromycin supplemented medium after 2 months of growth. (C) Close-up of embryos showing growth in various stages of development as well as emergence of secondary embryos. (D) Multiplication of transgenic shoots on banana shoot multiplication medium. (E) Rooting of transgenic banana plants on banana rooting medium containing NAA(1mg/l). (F) Rooted banana plants were hardened in a green house for GUS analysis. (PDF) [file pone.0192852.s003.pdf]

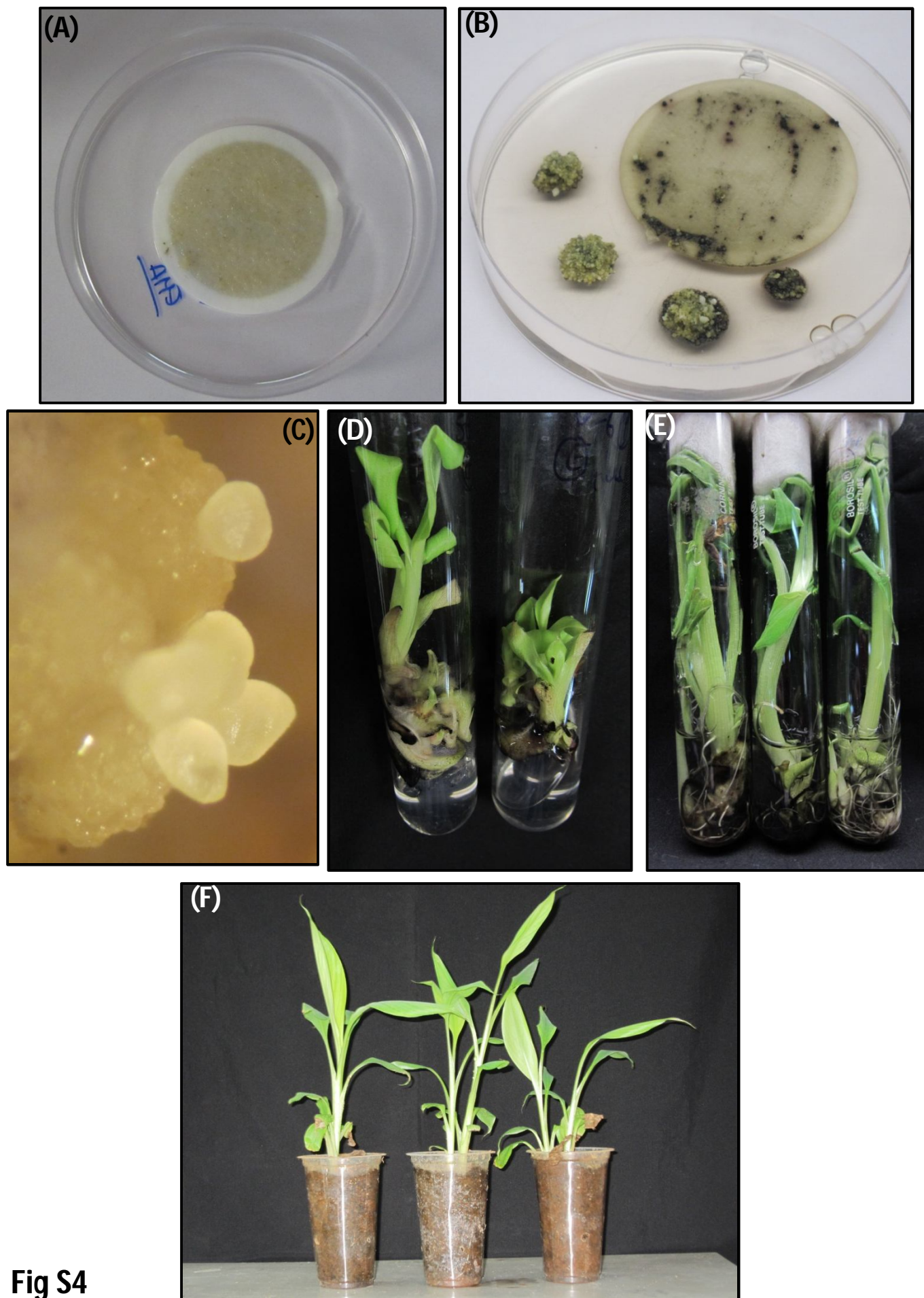

Fig S4

Supplement: S4 Fig — (A) Embryogenic cells of banana cultivar Rasthali growing on banana embryo development medium supplemented with hygromycin (5mg/l) after transformation with pCAMBIA1301- PMusaVND6:: GUS. (B) White and opaque embryos developed from continuous culturing of the transformed embryogenic cells on embryo development medium. (C) Developing embryos appeared globular to torpedo shaped in close-up. (D) Transformed banana shoots were multiplied on shoot multiplication medium to generate multiple lines. (E) Rooting of transgenic banana plants on banana rooting medium containing NAA(1mg/l). (F) Rooted banana plants were hardened in a green house for GUS analysis. (PDF) [file pone.0192852.s004.pdf]

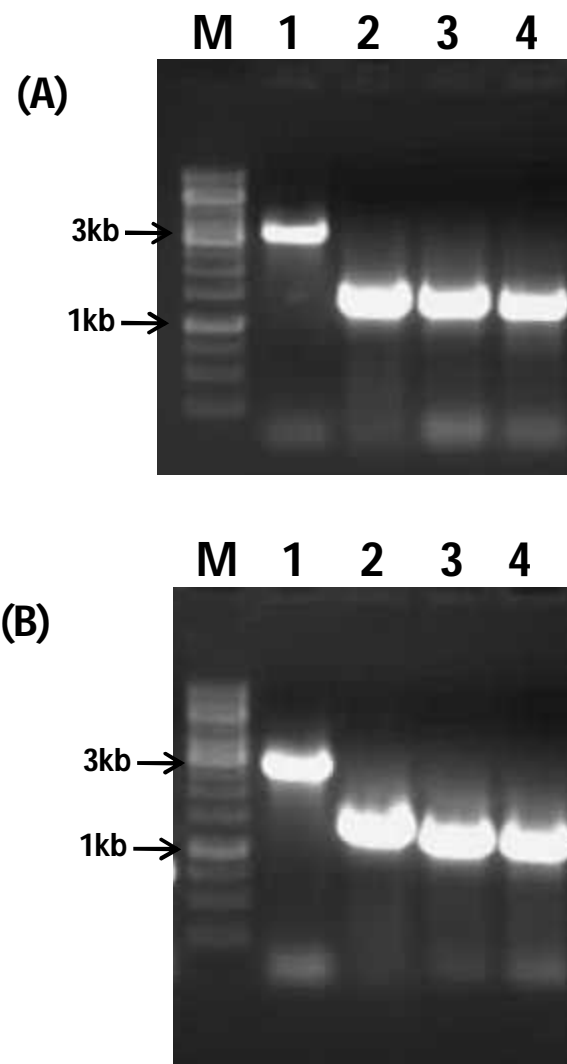

Fig S5

Supplement: S5 Fig — (A) PCR analysis of banana plants harboring PMusaVND7::GUS. (B) PCR analysis of banana plants harboring PMusaVND6::GUS. Two different primer pairs were utilized in PCR analysis. 1: FP in either PMusaVND7 or PMusaVND6 and RP in GUS; 2–4:FP and RP in either PMusaVND7 or PMusaVND6 2–4: analysis of three independent transgenic banana lines. M: 1 KB DNA ladder. 1kb and 3 kb band size are indicated. (FP: forward primer; RP: reverse primer). (PDF) [file pone.0192852.s005.pdf]

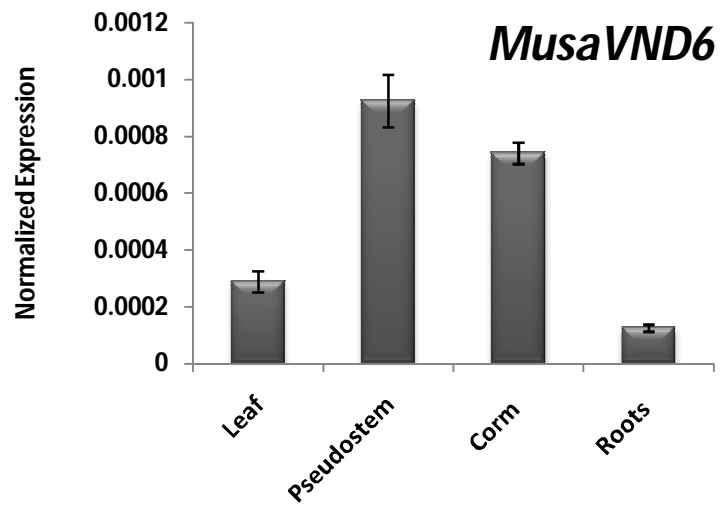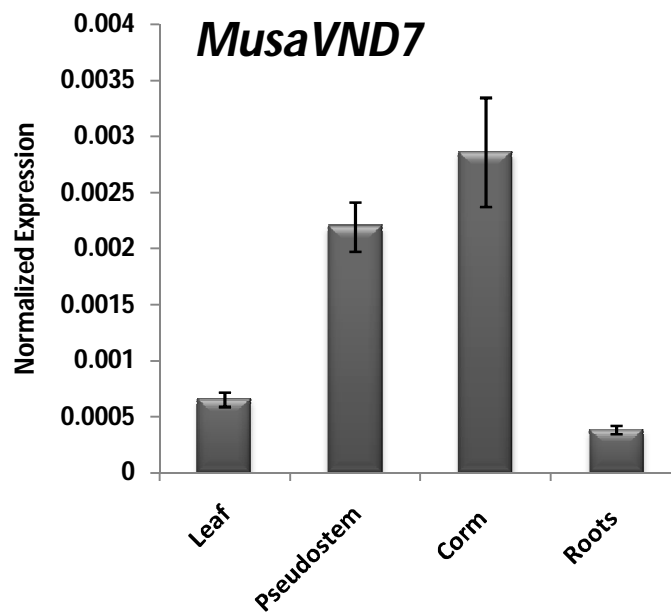

**Fig S6**

Supplement: S6 Fig — Transcript level of MusaVND6 and MusaVND7 in tissue of different organs of wild type banana was analyzed by quantitative RT-PCR. Expression of VND6 and VND7 in different tissue types is shown after normalization of the data by the expression of banana EF1α. Data was represented as mean±SD of three replications. (PDF) [file pone.0192852.s006.pdf]
